# HIGH-FREQUENCY failure of combination antiretroviral therapy in paediatric HIV infection is associated with unmet maternal needs causing maternal NON-ADHERENCE

**DOI:** 10.1016/j.eclinm.2020.100344

**Published:** 2020-05-08

**Authors:** Jane R. Millar, Nomonde Bengu, Rowena Fillis, Ken Sprenger, Vuyokazi Ntlantsana, Vinicius A. Vieira, Nisreen Khambati, Moherndran Archary, Maximilian Muenchhoff, Andreas Groll, Nicholas Grayson, John Adamson, Katya Govender, Krista Dong, Photini Kiepiela, Bruce D. Walker, David Bonsall, Thomas Connor, Matthew J. Bull, Nelisiwe Nxele, Julia Roider, Nasreen Ismail, Emily Adland, Maria C. Puertas, Javier Martinez-Picado, Philippa C. Matthews, Thumbi Ndung'u, Philip Goulder, the Ucwaningo Lwabantwana Consortium

**Affiliations:** a HIV Pathogenesis Programme, The Doris Duke Medical Research Institute, University of KwaZulu-Natal, Durban, South Africa; b Department of Paediatrics, University of Oxford, Oxford, United Kingdom; c Umkhuseli Innovation and Research Management, Pietermaritzburg, South Africa; d Department of Medicine, University of KwaZulu-Natal, Durban, South Africa; e Department of Paediatrics, University of KwaZulu-Natal, Durban, South Africa; f Max von Pettenkofer Institute, Virology, National Reference Center for Retroviruses, Faculty of Medicine, LMU München, Munich, Germany; g German Center for Infection Research (DZIF), Partner site Munich, Germany; h TU Dortmund University, Department of Statistics, Vogelpothsweg 87, 44227 Dortmund; i Africa Health Research Institute (AHRI), Durban, South Africa; j Ragon Institute of MGH, MIT, and Harvard, Cambridge, MA, United States; k Massachusetts General Hospital, Boston, Massachusetts, United States; l South African Medical Research Council, Durban 4001, SC Africa; m Wits Health Consortium, Johannesburg 2193, SC Africa; n Institute for Medical Engineering and Sciences and Department of Biology, Massachusetts Institute of Technology, Cambridge MA 02139, United States; o Howard Hughes Medical Institute, Chevy Chase MD 20815, United States; p Oxford Big Data Institute, Li Ka Shing Centre for Health Information and Discovery, Nuffield Department of Medicine, University of Oxford, Oxford, United Kingdom; q Cardiff University School of Biosciences, The Sir Martin Evans Building, Cardiff University, Cardiff, United Kingdom; r Pathogen Genomics Unit, Public Health Wales Microbiology Cardiff, University Hospital of Wales, Cardiff, United Kingdom; s Department of Infectious Diseases, Ludwig-Maximilians-University, Munich; t AIDS Research Institute IrsiCaixa, Badalona, Spain; u University of Vic-Central University of Catalonia (UVic-UCC), Vic, Spain; v Catalan Institution for Research and Advanced Studies (ICREA), Barcelona, Spain; w Nuffield Department of Medicine, University of Oxford, Oxford, United Kingdom; x Department of Microbiology and Infectious Diseases, Oxford University Hospitals NHS Foundation Trust, Oxford, United Kingdom; y Oxford BRC, John Radcliffe Hospital, Oxford, United Kingdom; z Max Planck Institute for Infection Biology, Berlin, Germany

**Keywords:** Hiv, Hiv cure, Paediatric hiv, Mother-to-child transmission

## Abstract

**Background:**

Early combination antiretroviral therapy (cART) reduces the size of the viral reservoir in paediatric and adult HIV infection. Very early-treated children may have higher cure/remission potential.

**Methods:**

In an observational study of 151 *in utero* (IU)-infected infants in KwaZulu-Natal, South Africa, whose treatment adhered strictly to national guidelines, 76 infants diagnosed via point-of-care (PoC) testing initiated cART at a median of 26 h (IQR 18–38) and 75 infants diagnosed via standard-of-care (SoC) laboratory-based testing initiated cART at 10 days (IQR 8–13). We analysed mortality, time to suppression of viraemia, and maintenance of aviraemia over the first 2 years of life.

**Findings:**

Baseline plasma viral loads were low (median 8000 copies per mL), with 12% of infants having undetectable viraemia pre-cART initiation. However, barely one-third (37%) of children achieved suppression of viraemia by 6 months that was maintained to >12 months. 24% had died or were lost to follow up by 6 months. Infant mortality was 9.3%. The high-frequency virological failure in IU-infected infants was associated not with transmitted or acquired drug-resistant mutations but with cART non-adherence (plasma cART undetectable/subtherapeutic, *p*<0.0001) and with concurrent maternal cART failure (OR 15.0, 95%CI 5.6–39.6; *p*<0.0001). High-frequency virological failure was observed in PoC- and SoC-tested groups of children.

**Interpretation:**

The success of early infant testing and cART initiation strategies is severely limited by subsequent cART non-adherence in HIV-infected children. Although there are practical challenges to administering paediatric cART formulations, these are overcome by mothers who themselves are cART-adherent. These findings point to the ongoing obligation to address the unmet needs of the mothers. Eliminating the particular barriers preventing adequate treatment for these vulnerable women and infants need to be prioritised in order to achieve durable suppression of viraemia on cART, let alone HIV cure/remission, in HIV-infected children.

**Funding:**

Wellcome Trust, National Institutes of Health.

**Research in context**

**Evidence before this study**

We searched PubMed up to May 31, 2019 for original and review articles, on HIV-infected infants; the timing and outcome of treatment, by searching the terms “HIV”, “antiretroviral therapy”, “infant treatment” and “outcome” or “suppression” or “early”, not limited by language. It has been well established that for perinatally HIV-infected infants, early cART initiation reduces morbidity, mortality, and viral reservoir size, suggesting the potential for cure/remission in this group. A recent study showed that cART initiation within the first 48 h of life for IU-infected infants is feasible in South Africa. However, for reasons unknown, it did not improve mortality, retention in care, or viral suppression rates in infancy.

**Added value of this study**

We studied a large, contemporary cohort of IU-infected infants in KwaZulu-Natal, South Africa. Pre-cART plasma HIV RNA and DNA viral loads were low. However, cART failure rates were high. In the subset of children tested, this was not due to transmitted or acquired cART resistance. High-frequency cART failure was due to cART non-adherence in the children.

**Implications of all the available evidence**

Where antenatal cART is available, IU-MTCT occurs in a vulnerable subset of mothers for whom cART self-administration, and administration to their infected child, is difficult to achieve. In order to maximise health in perinatally infected children, as well as the high potential for HIV cure/remission, early infant diagnosis and cART initiation alone is insufficient without meeting the socioeconomic needs of transmitting mothers. There is an urgent need to address the particular challenges facing mothers of HIV-infected infants, and at the same time to explore long-acting therapeutic options to lessen the medication burden for effective anti-HIV treatments for children.

Alt-text: Unlabelled box

## 1 Introduction

Initiation of combination antiretroviral therapy (cART) early in HIV infection reduces the size of the viral reservoir, estimated by the frequency of HIV DNA in peripheral blood cells, both in paediatric and adult infection [1], [2], [3]. Low proviral DNA load is the most consistent correlate of cure/remission [3]. However, additional factors other than limiting the viral reservoir via early cART initiation, including immune, host genetic, virological and environmental factors, are likely to influence the ability to achieve cure/remission [2,4,5].

There is some albeit limited evidence that the potential to achieve cure/remission may be greatest in children following *in utero* (IU) infection [1]. Point-of-care (PoC) testing and cART initiation can be implemented very soon after birth [6], and, therefore, very soon after infection, since most IU infections arise late in pregnancy [7]. Furthermore, the tolerogenic immune response in early life mitigates against immune activation [4]. This contrasts with the aggressive immune response to HIV in adults which, although better at suppressing viral replication, also results in detrimental immune activation and inflammation [8], accelerating establishment of the viral reservoir.

Several anecdotal cases of cure/remission in early cART-treated children further support the notion that initiation of cART within the first days of life might pave the way to achieving cure/remission in a substantial proportion of those treated [9], [10], [11]. However, a recent study in South Africa suggests that outcomes in early-treated IU-infected infants are not successful [6]. To further explore the factors, including cART non-adherence and drug resistance, underlying success or failure of early cART in this group, we studied IU-infected infants enroled from hospitals in KwaZulu-Natal, South Africa where the prevention of mother-to-child transmission (PMTCT) programme has been very effective. IU-MTCT rates have fallen from 7% to 0.5% since the provision of cART to all HIV-infected mothers during pregnancy [1,12] but HIV seroprevalence in mothers attending antenatal clinics has continued to rise even since the introduction of cART in 2004, and approaches 40% [13]. This setting therefore provided the opportunity to evaluate the success of early cART on outcome following IU-infection.

## 2 Methods

### 2.1 Study design and participants

Ucwaningo Lwabantwana (meaning ‘Learning from Children’) is an ongoing observational prospective cohort study designed to determine the feasibility of very early (<48 h of life) cART initiation for IU HIV-infected infants. In 2015, the study began to identify IU HIV-infected infants from four secondary-level hospitals in KwaZulu-Natal, South Africa. In this setting, there is lifelong cART for pregnant women and routine infant birth HIV total nucleic acid (TNA) PCR.

Baseline results from this current cohort were compared to IU HIV-infected infants from a historical cohort set in Durban in KwaZulu-Natal in 2002–2005 (PEHSS, Paediatric Early HAART and Strategic Treatment Interruption Study) [14,15]. At that time, there was no ART available to treat adults for HIV infection and perinatal ART prophylaxis was single dose nevirapine (NVP) for both mother and infant. Raw data from the PEHSS cohort were available for all 50 mother/IU HIV-infected infant pairs at baseline.

### 2.2 Ethics

These studies were approved by the Biomedical Research Ethics Committee of the University of KwaZulu-Natal and the Oxfordshire Research Ethics Committee. Written informed consent for the infant and mother's participation in the study was obtained from the mother or infant's legal guardian.

### 2.3 Study definitions

**High-risk mother:** one or more of the following criteria during pregnancy; maternal seroconversion, <4 weeks of maternal cART prior to delivery, suboptimal cART adherence by history or a documented plasma viral load >1000 HIV RNA copies per mL.

**Maternal seroconversion:** a documented negative rapid HIV-1 antibody test in pregnancy followed by a positive result later in pregnancy or at delivery.

**cART non-adherence:** missing three or more consecutive cART doses.

***In utero* HIV-infected:** an infant with a positive or indeterminate TNA PCR taken <48 h of age that was later confirmed positive via further testing from a separate blood samples i.e. in all cases diagnosis required two or more positive nucleic acid tests.

**Viral suppression:** Plasma HIV RNA level below the limit of detection (<20 or <100 HIV RNA copies per mL depending on sample volume) on one occasion.

**Viral rebound:** Plasma HIV RNA >1000 copies per mL on one occasion or two consecutive measurements >100 HIV RNA copies per mL following viral suppression.

**Loss to follow-up:** an infant that did not return to the study site for ≥6 months despite active tracing.

### 2.4 Procedures

For the Ucwaningo Lwabantwana study, HIV-positive mothers were screened after delivery to assess risk of IU HIV transmission. Infants of high-risk mothers were tested for HIV-1 as soon as possible after birth using PoC TNA PCR on whole blood (GeneXpert Qualitative HIV-1 PCR, Cepheid, Sunnyvale, CA, USA) or where PoC testing was not available, by overnight plasma HIV RNA viral load measurement (Nuclisens EasyQ v2.0 HIV-1 RNA PCR, bioMérieux, Marcy l'Etoile, France). The results of these tests were binary with no indeterminate range.

Infants from the same hospitals who either declined PoC testing or who were discharged before they could be tested, and those born at the hospitals’ local clinics with a positive or indeterminate result from the standard-of-care (SoC) laboratory-based dried blood spot TNA PCR (COBAS AmpliPrep /COBAS TaqMan HIV-1 Qualitative PCR v2, Roche Molecular Diagnostics, Basel, Switzerland) at birth were also enroled. Turnaround time for the SoC test result was approximately 4–7 days. In all cases diagnosis required two or more positive HIV nucleic acid tests. Eligibility criteria for enrolment via any method of testing included an IU HIV-infected infant initiated on cART ≤21 days of life.

All HIV exposed infants were given prophylactic ART as soon as possible after birth, consisting of at least daily NVP, and additional twice daily zidovudine (AZT) for infants deemed high risk of HIV-1 infection, in accordance with the South African National PMTCT Guidelines. After the first positive infant HIV-1 test result was received, blood for confirmatory testing and baseline measurements was taken and cART consisting of twice daily NVP, AZT and lamivudine (3TC) was initiated as per local guidelines. This neonatal regimen was switched to ritonavir-boosted lopinavir (LPVr), 3TC and abacavir (ABC) at 42 weeks corrected gestational age or at 1 month of age.

Mother and infant follow-up occurred monthly for 6 months then 3 monthly. At each visit, blood was drawn for CD4+ *T* cell quantification, plasma viral load (HIV-1 RNA PCR, NucliSens), and storage of peripheral blood mononuclear cell (PBMC) and plasma.

During follow-up, additional support was provided for the caregivers, including regular one-on-one counselling sessions from trained HIV counsellors in the local language (isiZulu), paid transport to clinic, appointment reminders via phone, family support groups and home visits where required.

Stored PBMC and plasma samples were used for additional confirmatory testing, and where available, for the quantification of drug resistance mutations (DRMs) by Next Generation sequencing methods, HIV DNA by droplet digital PCR (ddPCR; BioRad, Hercules, California, USA), and plasma ART levels by high performance liquid chromatography coupled to tandem mass spectrometry (Supp. Methods).

### 2.5 Analysis and outcomes

To assess the feasibility and impact of very early cART initiation in this current cohort, Ucwaningo Lwabantwana, we considered outcomes for the whole cohort of IU HIV-infected infants together and then compared two subgroups of IU-infected infants according to the timing of test they were first identified by: the PoC and SoC groups. Comparisons included: time to cART initiation, maternal and infant baseline characteristics, time to viral suppression, viral rebound rates, mortality, loss to follow-up and viral suppression rates at 12 and 24 months. To assess the influence of drug resistance on virological outcomes, DRMs were quantified and plasma ART levels were measured to evaluate potential non-adherence to treatment. To assess the impact of the current PMTCT programme, these infants’ baseline results were also compared to those infants from the historical PEHSS cohort.

### 2.6 Statistical analysis

Clinical and laboratory results were described using absolute numbers, percentages, medians and interquartile range (IQR). Comparisons were performed using the Chi-Squared and Fisher's exact tests for categorical variables and the Mann-Whitney U test for continuous variables. Mortality rate, viral suppression and viral rebound were calculated in univariate analyses using Kaplan-Meier curves and groups were compared using the Log-Rank test. Because of the large number (25) of candidate mother-child variables at baseline, predictors of mortality, viral suppression and viral rebound were analysed in multivariate analyses using a penalised Cox regression model, where the set of relevant predictors was selected by the LASSO penalty approach [16] and the optimal penalty parameter *ξ* was determined via 10-fold cross validation using the glmnet R package [17]. A sensitivity analysis was carried out by using an alternative definition of viral rebound; the date the plasma viral load exceeded HIV RNA 1000 copies per mL. The relationship between maternal and infant viral loads was calculated using Spearman's non-parametric correlation. All p-values were two-sided. Calculations and graphs were performed using R Software v3.6.1 and GraphPad Prism v8 respectively.

## 3 Role of the funding source

The funders of this study had no role in study design, data collection, data analysis, data interpretation, or writing of the report. The corresponding author had full access to all the data in the study and had final responsibility for the decision to submit for publication.

## 4 Results

**Fig. 1:**
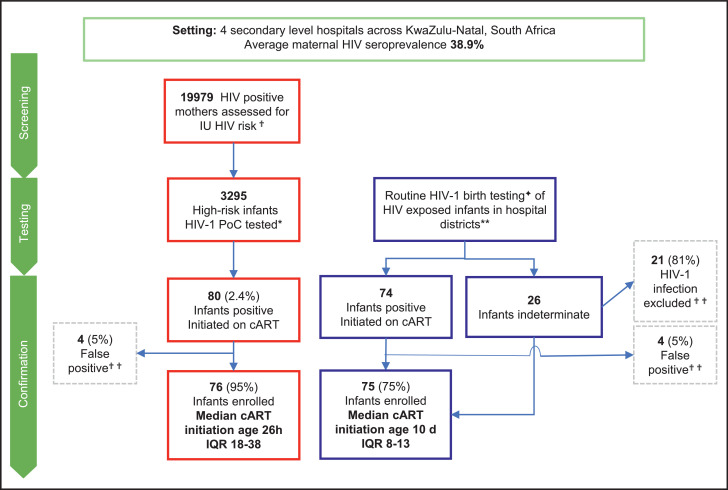
Study Profile. ^✟^ High risk; during pregnancy the mother either seroconverted, had <4 weeks cART, poor cART adherence by history or a documented plasma viral load >1000 HIV RNA copies per mL. *Tested with point-of-care (PoC) whole blood GeneXpert (GXP) HIV-1 Qualitative total nucleic acid (TNA) PCR (Cepheid); but when not available an overnight HIV RNA test; plasma Nuclisens EasyQ v2·0 HIV-1 RNA PCR (bioMérieux). ^✦^Laboratory-based dried blood spot TNA PCR (CAP/CTM HIV-1 Qualitative Test v2 Roche) **iLembe, uMgungundlovu, eThekwini and uThungulu districts. ^✟ ✟^Confirmatory testing included; TNA PCR via CAP/CTM or GXP, HIV RNA PCR via Nuclisens or in selected cases more sensitive methods. IU: *in utero*, cART: combination antiretroviral therapy.

Between July 01, 2015 and January 31, 2019, 151 IU-infected infants were enroled onto the Ucwaningo Lwabantwana study (Fig 1, Table 1). In all 151 cases, infant ART prophylaxis was initiated within minutes of birth prior to diagnosis of IU infection. Two-thirds of infants (103/151, 68%) received both NVP and AZT, the remaining one-third (48/151, 32%) receiving NVP or AZT only. Of these 151 IU-infected infants, 76 were identified through PoC (*n* = 70) or overnight viral load (*n* = 6) testing of infants, and cART was initiated at a median age of 26 h (IQR 18–38). A further 75 infants were diagnosed through SoC testing, in whom cART was initiated at a median age of 10 days (IQR 8–13). ART prophylaxis did not differ significantly between the PoC and SoC groups (Table 1).

**Table 1:** Demographic and clinical information of mother and *in utero* HIV-infected infant pairs at baseline.

	All		Point-of-care Tested		Standard-of-care Tested		
	(*n* = 151)		(*n* = 76)		(*n* = 75)		
MOTHER		IQR/%		IQR/%		IQR/%	P-value of PoC vs SoC
Median maternal age (years)	24.7	(21.5–30.0)	24.1	(21.4–27.9)	26.3	(21.4–27.9)	0.088
Timing of maternal HIV infection	..	..	..	..	..	..	..
*Chronic; vertical*	6	4.0%	3	4.0%	3	4.0%	0.41
*Chronic; horizontal*	36	23.8%	14	18.4%	22	29.3%	..
*Acute*	47	31.1%	27	35.5%	20	26.7%	..
*Unknown*	62	41.1%	32	42.1%	30	40.0%	..
Previous MTCT	7	4.6%	4	5.3%	3	4.0%	1.0
Median number of other children	1	(0–2)	1	(0–2)	1	(0–2)	0.25
Timing of maternal cART initiation	..	..	..	..	..	..	..
*Pre-pregnancy*	31	20.5%	13	17.1%	18	24.0%	0.33
*During pregnancy >12 weeks prior to delivery*	50	33.1%	26	34.2%	24	32.0%	..
*During pregnancy <12 weeks prior to delivery*	33	21.9%	16	21.1%	17	22.7%	..
*During labour or at delivery*	20	13.2%	14	18.4%	6	8.0%	..
*Postnatally*	17	11.3%	7	9.2%	10	13.3%	..
Maternal cART regimen at enrolment	..	..	..	..	..	..	..
*1st line (NNRTI)*	146	96.7%	75	98.7%	71	94.7%	0.30
*2nd line (PI)*	3	2.0%	1	1.3%	2	2.7%	..
None*	2	1.3%	0	0%	2	2.7%	..
Self-reported antenatal cART non-adherence or refusal†	63	55.3%	30	54.5%	33	55.9%	0.69
Median plasma viral load (HIV RNA copies per mL)	4400	(520–40 500)	12 500	(1400–160 000)	2200	(133–12 500)	0.0002
Median absolute CD4+ *T* cell count (cells/μL)	452	(318–637)	442	(298–604)	462	(348–641)	0.45
Median CD4:CD8	0.46	(0.31–0.69)	0.44	(0.30–0.62)	0.5	(0.34–0.75)	0.10
**INFANT**	..	..	..	..	..	..	..
Sex	..	..	..	..	..	..	..
*Male*	55	36.4%	20	26.3%	35	46.7%	0.011
*Female*	96	63.6%	56	73.7%	40	53.3%	..
Median Gestation at birth (weeks)‡	38	(36–39)	37	(36–39)	38	(36–40)	0.24
Median Birth weight (kg)	2.82	(2.40–3.14)	2.81	(2.36–3.09)	2.86	(2.50–3.20)	0.44
Small for gestational age‡§	32	21.2%	16	21.1%	16	21.3%	1.00
Exclusively breastfed	120	79.5%	69	90.8%	51	68.0%	0.0011
Neonatal admission¶	46	30.5%	23	30.3%	23	30.7%	1.00
Infant ART prophylaxis	151	100%	76	100%	75	100%	1.0
*Nevirapine only*	47	31.1%	26	34.2%	21	28.0%	0.45
*Zidovudine only*	1	0.7%	0	0.0%	1	1.3%	..
*Nevirapine and Zidovudine*	103	68.2%	50	65.8%	53	70.7%	..
Median plasma viral load (HIV RNA copies per mL)	8350	(1500–67 500)	29 500	(3325–342 500)	3350	(300–22 500)	<0.0001
Median absolute CD4+ *T* cell count (cells per μL)	2035	(1263–2700)	1682	(1012–2299)	2444	(1811–3147)	<0.0001
Median CD4+ *T* cell percentage	43	(35–52)	39	(31.8–49.5)	46	(37–53)	0.011
Median CD4:CD8	1.86	(1.13–2.84)	1.54	(1.05–2.65)	2.08	(1.36–3.21)	0.043
Standard-of-care TNA PCR birth test result	..	..	..	..	..	..	..
*Positive*	136	90.1%	66	86.8%	70	93.3%	0.25
*Indeterminate*	13	8.6%	8	10.5%	5	6.7%	..
*Negative*	2	1.3%	2	2.6%	0	0%	..
Age of cART initiation (hours or days)	4d	(26h–10d)	25.6h	(17.7–37.9 h)	10d	(8–13d)	<0.0001

MTCT: mother-to-child transmission, NNRTI: non-nucleoside reverse transcriptase inhibitor, PI: protease inhibitor, cART: combination antiretroviral therapy.

⁎ Two mothers had cART initiation delayed beyond enrolment while TB infection was excluded.

† Of those who initiated cART prior to labour.

‡ 6 infants had unknown gestational age at birth.

§ As classified by InterGrowth 21st charts.

¶ Direct to neonatal ward or to the paediatric ward aged ≤ 28 days of life.

With respect to timing of maternal infection, 31% (47/151) of the IU-infected infants were born to mothers designated as seroconverting during the pregnancy. This included 41 mothers who had a negative HIV test during pregnancy followed subsequently by a positive test; and 6, in whom the estimated seroconversion date, midway between last negative test and first positive test, was also during pregnancy. In 28% (42/151) of cases, the infants were born to mothers known to have been HIV-infected prior to conception, including 6 who were themselves infected via MTCT (Table 1). The remaining 62/151 (41%) mothers’ first HIV test during the pregnancy was HIV-positive and the timing of infection was unknown (Table 1). Due to lacking antenatal care, or seroconversion late in pregnancy, cART had not been initiated in 25% (37/151) of mothers prior to the onset of labour. amongst the 75% (114/151) of mothers who initiated cART prior to the onset of labour, 63 (55%) self-reported poor cART adherence, of which nine (8%) omitted all doses. Timing of maternal infection and maternal cART initiation did not differ between the PoC and SoC groups (Table 1).

The majority of IU-infected infants were healthy, term babies (median gestation 38 weeks [IQR 36–39], median birth weight 2.82 kg [IQR 2.4–3.14]) (Table 1). However, 32/151 (21%) were small-for-gestational age (birth weight <10th centile on InterGrowth 21st charts [18] and 46/151 (31%) of infants required hospital admission in the first 28 days of life. As previously reported [19], the number of female IU-infected infants exceeded that of males (96 vs 55). For reasons that are unclear, the sex difference was substantially greater in the PoC group (females:males 2.8:1) compared to the SoC group (females:males 1.1:1; *p* = 0.011) (Table 1).

**Fig. 2:**
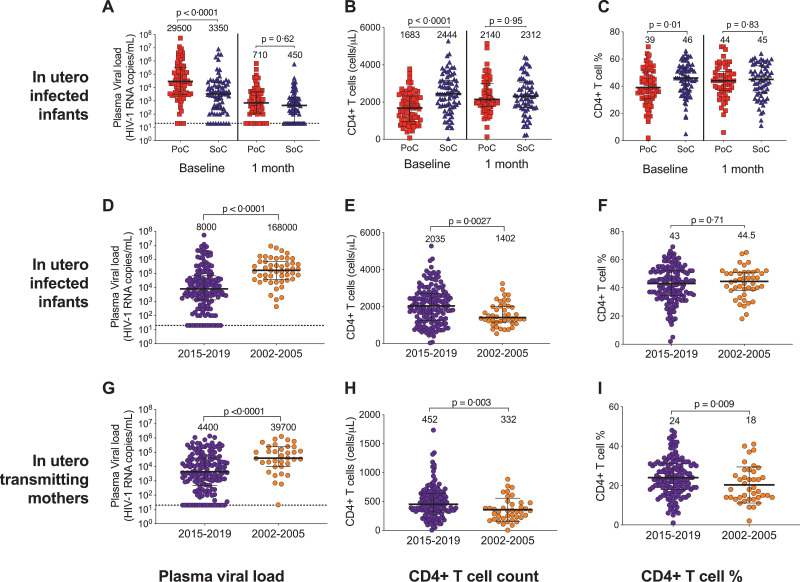
Effects of very early ART initiation and universal maternal cART on baseline plasma HIV viral load and CD4+ *T* cell count. (A) Plasma HIV RNA viral load, (B) absolute CD4+ *T* cell count and (C) CD4+ *T* cell percentage of the IU HIV-infected infants at baseline (<21 days of life, prior to combination antiretroviral therapy (cART) initiation) (left) and at 1 month of age (right), where PoC were diagnosed using a point-of-care HIV TNA PCR or overnight RNA PCR test and treated very early (median age 26 h) and SoC were diagnosed by the standard-of-care laboratory-based HIV PCR test and treated early (median age 10 days). (D) Plasma HIV RNA viral load, (E) absolute CD4+ *T* cell count and (F) CD4+ *T* cell percentage of the IU HIV-infected infants within 21 days of birth where 2015–2019 is the current cohort (Ucwaningo Lwabantwana) and 2002–2005 are the cohort from the same population prior to universal antenatal cART (PEHSS). (G) Plasma HIV RNA viral load, (H) absolute CD4+ *T* cell count and (I) CD4+ *T* cell percentage of the mothers of the IU HIV-infected infants shown in figure d-F, within a month of delivery. Median values and interquartile range displayed. All p-values calculated using the Mann-Whitney U test.

The first (‘baseline’) measurements of plasma viral load and CD4+ *T* cell count in the PoC and SoC groups were taken, respectively, at a median of 1 day and 10 days of age, and thus after 1 and 10 days of prophylactic ART (NVP alone or AZT/NVP). As expected, therefore, at baseline, the SoC infant group had significantly lower plasma viral loads than the PoC group (median 3350 vs 29 500 HIV RNA copies per mL; *p*<0.0001), higher CD4+ *T* cell counts (median 2444 vs 1682 cells per uL; *p*<0.0001) and higher CD4+ *T* cell percentage, (median 46% versus 39%, *p* = 0.01; Fig 2A–C). However, by 1 month of age, differences between the PoC and SoC groups in plasma viral load, absolute CD4+ *T* cell count and CD4+ *T* cell percentage had disappeared (Fig 2A–C).

Baseline infant plasma HIV viral loads were significantly lower and absolute CD4+ *T* cell counts significantly higher in the current Ucwaningo Lwabantwana cohort of 151 IU-infected infants compared to the 2002–2005 PEHSS cohort of 50 IU-infected infants, also in KwaZulu-Natal, South Africa (Fig 2D–F), when cART was not available to mothers during pregnancy. Similarly, in the IU transmitting mothers, plasma viral loads were lower in the current Ucwaningo Lwabantwana cohort compared to the PEHSS cohort (4400 vs 39 700 RNA copies per mL *p*<0.0001, Fig. 2G). In the 23 mothers from the Ucwaningo Lwabantwana cohort who had no ART prior to labour and plasma viral load measurement within 1 week of delivery, plasma viral load was similar to the PEHSS cohort (16 000 vs 39 700 HIV RNA copies per mL, *p* = 0.09, not shown). It is noteworthy that as many as 19/151 (12.5%) of the Ucwaningo Lwabantwana cohort of IU-infected infants had undetectable plasma viral loads (<20 HIV RNA copies per mL or <100 HIV RNA copies per mL) at baseline, consistent with the notion that, in the majority of IU-infected infants, there is significant intrauterine ART exposure transplacentally.

**Fig. 3:**
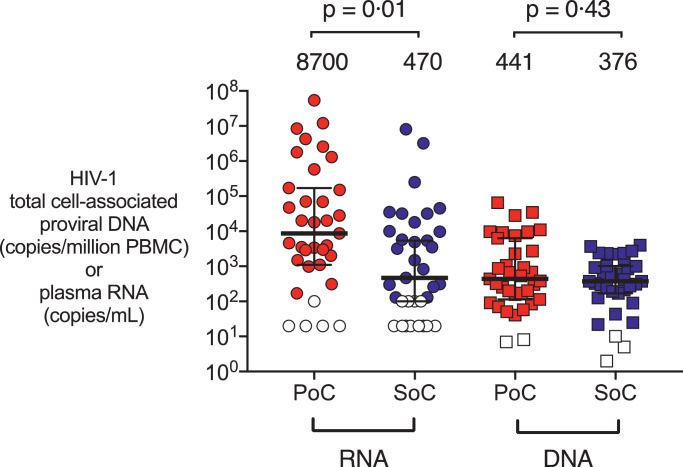
Baseline infant proviral HIV DNA levels compared to plasma RNA. Baseline infant plasma HIV RNA levels (copies per mL) (left) and proviral HIV DNA levels (copies per million peripheral blood mononuclear cells (PBMC)) as measured by droplet digital PCR (right) for a subset of infants at baseline, a comparison for PoC, diagnosed by point-of-care test and treated very early (median time 26 h) and SoC, those diagnosed by standard-of-care laboratory-based test and treated early (median time 10 days). Median values and interquartile range displayed. Open symbols are below the limit of detection (20 or 100 copies per mL for RNA and variable for each individual test for DNA (2–10 copies per million PBMC). P-values calculated using the Mann-Whitney U test.

To investigate further whether delaying cART initiation to day 10 compared to initiation on day 1 of life might affect the size of the viral reservoir in the PoC and SoC groups of infants at the time of cART initiation, HIV DNA viral loads were measured by ddPCR in a subset of 35 infants, selected according to sample availability. Even though the plasma HIV RNA loads were 1.2–1.3 log_10_ higher in the PoC group, HIV DNA viral loads measured at the same timepoint showed minimal difference between the groups (<0.07 log_10_ copies HIV DNA per million PBMC; *p* = 0.43, Fig 3). Together with the data showing a lack of difference in plasma viral loads between the PoC and SoC groups at 1 month of age, these findings suggest that SoC testing and cART initiation at median 10 days does not bring any significant disadvantage compared with PoC testing and cART initiation at median 26 h in a setting where prophylactic ART is initiated in all babies born to HIV-positive mothers within minutes of birth and ART exposure commonly occurs *in utero*.

**Fig. 4:**
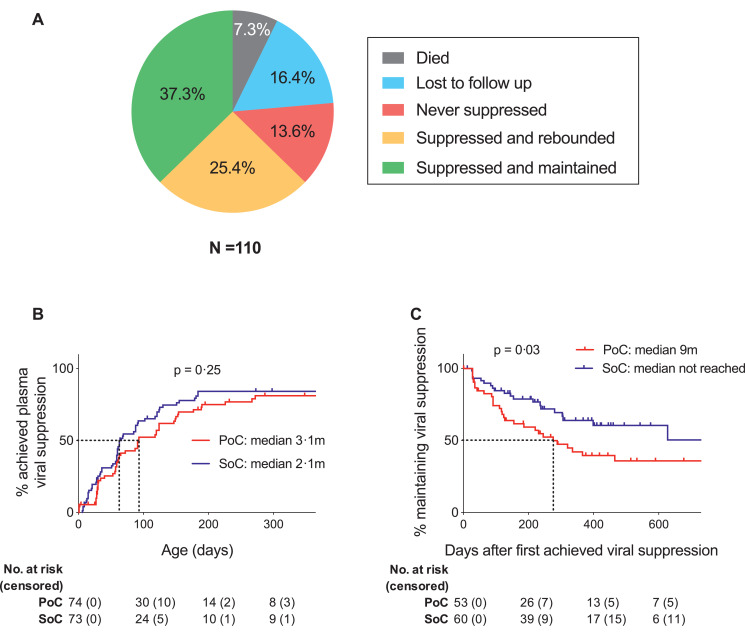
Infant outcomes: retention, mortality and plasma viral suppression. (A) Proportions of infants with the opportunity for 12 months follow-up (excluding 4 infants who were withdrawn by the investigators) who died, were lost to follow-up, those who did not achieve plasma viral suppression by 6 months of age, those who did achieve plasma viral suppression by 6 months of age but had rebounded by 12 months of age, and those who achieved plasma viral suppression by 6 months of age and maintained suppression to at least 12 months of age. (B) Kaplan-Meier analysis of time to plasma viral suppression; a comparison of PoC and SoC groups. PoC infants were diagnosed using a point-of-care HIV TNA PCR or overnight RNA PCR test and treated very early (median time 26 h) and SoC are those diagnosed by the standard-of-care laboratory based HIV PCR test and treated early (median time 10 days). (C) Kaplan-Meier analysis of time to plasma viral rebound; a comparison of PoC and SoC groups.

To assess initial outcome in SoC and PoC-treated, IU-infected infants, we next evaluated the 114/151 infants from the Ucwaningo Lwabantwana cohort who were born ≥12 months prior to the time of analysis. Excluding four infants (two PoC- and two SoC-tested) who transferred out in the first week of life to receive care at external clinics, 26 of the remaining 110 (24%) had either died (*n* = 8, 7.3%), or were lost to follow-up (*n* = 18, 16.4%) (Fig 4A). amongst the latter, six died subsequent to defaulting clinic appointments. Thus, in the cohort overall (*n* = 151), 15 infants died. The probability of death by age 3 months was 6.4% (95% CI 2.3–10.3%) and was 9.3% by 1 year (95% CI 4.4–14.1%; not shown) with a median age of death of 3.5 months. The multivariate analysis identified three independent predictors of mortality: low infant birthweight; neonatal hospital admission and high infant baseline plasma HIV RNA viral load (Supp. Tab. 1).

Of the infants retained on the study at 12 months of age, 15/110 (13.6%) had not achieved suppression of plasma viraemia on cART by 6 months and 28/110 (25.5%) had achieved only short-term suppression of viraemia, rebounding by 12 months (Fig 4A). Only 37.3% of the 110 IU-infected infants enroled had achieved and maintained suppression of viraemia on cART to ≥12 months of age. 40 out of the 84 infants on the study at 12 months were also analysed 24 months post-enrolment. 14/17 (82.4%) of those infants who had suppressed by 6 months and maintained suppression to 12 months, continued to maintain viraemic suppression to 2 years of age. In contrast, only 2/17 (12%) of those whom only had short term suppression between 6 and 12 months, and 0/6 of those who had not supressed by 6 months, achieved and maintained suppression to 2 years (Supp. Fig. 1).

In order to further investigate why sustained suppression of viraemia in early-treated infants on ART is so infrequent, we first compared the PoC and SoC groups. Although there was no difference in mortality or retention on the study at 12 months (*p* = 0.75, and *p* = 0.19, respectively), in the univariate analyses, infants in the SoC group tended to achieve suppression of viraemia earlier, and to maintain suppression of viraemia longer than those in the PoC group (Fig 4B–C, *p* = 0.25 and *p* = 0.03, respectively). These findings held true in the multivariate analyses, with a strong predictor at baseline for rebound being the PoC group compared with the SoC group, Supp Table 1. The strongest predictive factor at baseline for time to viral suppression was baseline infant plasma viral load and was not influenced by the age at which the infant initiated cART. Aside from SoC vs PoC group influencing time to infant viral rebound, longer duration of maternal cART in pregnancy and older infant gestational age at birth was also found to be associated with a faster rate of rebound, while older maternal age, male infants, and being admitted to hospital in the neonatal period was associated with a slower rate (Supp. Table 1). There was no significant change to the rate of viral rebound when relaxing the definition of viral rebound to a level >1000 HIV RNA copies per mL only (Supp Fig 2). High rates of treatment failure suggest either inferior cART adherence or reduced cART effectiveness due to drug resistant mutations (DRMs).

To investigate whether ART resistance might explain the high rates of virological failure on ART in the Ucwaningo Lwabantwana cohort, we first addressed the question of whether drug resistant mutations (DRMs) were transmitted from the mother to the child. Samples from a subset of infants (*n* = 14, selected according to sample availability) were deep sequenced for DRMs at baseline (median age 5.5 days [IQR 1–26 days], Table 2). amongst these 14 infants, seven achieved suppression of viraemia by 6 months of age, in three cases without any DRMs detected, and in the remainder with only one NNRTI DRM detected in the child's virus. amongst the seven infants who did not achieve suppression of viraemia by 6 months, four had no detectable DRMs and the remaining three had two or three NNRTI DRMs at baseline corresponding to high-level resistance to NVP. Given the short duration the infants receive NVP-based cART before switching to protease inhibitor (PI)-based cART and the absence of any PI DRMs at baseline, these data are consistent with ART non-adherence rather than transmitted DRMs being the cause of virological failure in these children.

**Table 2:** Mother and infant transmitted drug resistance genotype and phenotype.

						Genotype (DRMs)			Resistance level		
Pair		Days post delivery	Duration cART (days)	Duration maternal cART at delivery	Plasma viral load (RNA copies per mL)	NNRTI	NRTI	PI	Intermediate	High	Infant age at viral supp-ression (m)
**1**	**Mother**	1	1	0	6300	none	none	none	none	none	..
	**Child**	1	0	0	4600	none	none	none	none	none	1
**2**	**Mother**	9	150	141	100 000	none	none	none	none	none	..
	**Child**	9	0	141	830	none	none	none	none	none	1
**3**	**Child**	9	0	0	3400	K103N (100%)	none	none	none	EFV, NVP	2
**4**	**Mother**	1	>5 years	>5 years	13 000	K103N (77%), Y188L (25%)	none	none	none	EFV, NVP	..
	**Child**	1	0	>5 years	150 000	Y188L (99%)	none	none	none	EFV, NVP	2
**5**	**Child**	1	34	33	3900	none	none	none	none	none	5
**6**	**Mother**	12	17 years	17 years	2800	none	none	none	none	none	..
	**Child**	53	41	17 years	36 000	Y181C (98%)	none	none	EFV	NVP	5
**7**	**Child**	1	0	159	430 0000	K103N (100%)	none	none	none	EFV, NVP	6
**8**	**Mother**	1	0	0	1 100 000	none	none	none	none	none	..
	**Child**	28	28	28	53 000	none	none	none	none	none	5
**9**	**Mother**	18	8 years	8 years	3400	K103N (20%)	none	none	none	EFV, NVP	..
	**Child**	18	0	8 years	32 000	K103N (45%), V106A (29%), G190A (22%)	none	none	none	EFV, NVP	27
**10**	**Mother**	11	37	26	28 000	K103N (66%)	none	none	none	EFV, NVP	..
	**Child**	60	49	26	57 000	K103N (98%), V106A (28%), Y188C (22%)	none	none	none	EFV, NVP	>18
**11**	**Child**	90	88	245	450 000	K103N (86%), A98G (99%)	none	none	none	EFV, NVP	>18
**12**	**Mother**	2	4	2	16 000	none	none	none	none	none	..
	**Child**	2	0	2	1 500 000	none	none	none	none	none	LTFU at 1
**13**	**Mother**	0	203	203	770 000	none	none	none	none	none	..
	**Child**	0	0	203	2 900 000	none	none	none	none	none	died at 8
**14**	**Mother**	2	0	0	43 000	none	none	none	none	none	..
	**Child**	2	0	0	350 000	none	none	none	none	none	LTFU at 15

HIV genotype was determined using Next-Generation Sequencing methods for mother and infant pairs at baseline, subject to sample availability. Drug resistance mutation (DRM) prevalence shown in brackets. Resistance phenotype for the antiretroviral drugs commonly used in South Africa were determined from the Online Stanford Database. Median infant age at blood draw was 5.5 days (IQR 1–26 days). Participants listed in order of increasing time to infant plasma viral suppression (in months). No nucleoside reverse transcriptase inhibitor (NRTI) or protease inhibitor (PI) resistance mutations were found for any of the participants. NNRTI: non-nucleoside reverse transcriptase inhibitor, NVP: nevirapine, EFV: efavirenz.

We next investigated whether virological failure might have resulted from DRMs acquired in older infants. Samples were analysed from a second subset of infants (*n* = 16, selected according to sample availability) aged >6 months (median 12 months, IQR 8–15; median viral load 24 000 HIV RNA copies per mL) with virological failure, defined as a plasma RNA viral load of >1000 HIV RNA copies per mL. We also measured levels of antiretroviral drugs in these 16 plasma samples, since although ART detection reflects adherence only in the previous 24–48 h, ART non-detection unequivocally demonstrates non-adherence during this window (Table 3). In four subjects, viral suppression was never achieved, and no DRMs were detected in the viruses. Three of these subjects had no ART detectable, and one only 3TC, consistent with poor or no ART adherence. In the remaining 12 subjects, eight had high-level 3TC resistance. However, in nine of these 12, virological failure was matched by absence of cART detectable in the plasma, and six of nine infants who were not subsequently lost to follow up later suppressed viraemia on the same regimen of ABC/3TC/LPVr. This suggests that, in the absence of PI DRMs, adherence to this regimen can still achieve viral suppression.

**Table 3:** Infant drug resistance genotype and phenotype at 12 months.

			Genotype - DRMs			Resistance level				
Child	Age at blood draw (m)	Plasma viral load (HIV RNA copies per mL)	NNRTI	NRTI	PI	low	Intermediate	high	Subsequently suppressed?	Plasma ART detected
**1**	36	17 000	none	none	none	none	none	none	no	none
15*	9	15 000	none	none	none	none	none	none	no	3TC
**16**	15	24 000	none	none	none	none	none	none	no - LTFU	none
**17**	6	410 000	none	none	none	none	none	none	no - LTFU	none
**18**	12	350 000	K103N (88%)	none	none	none	none	EFV, NVP	yes	none
19*	9	2100	none	M184V	none	ABC	none	3TC	yes	LPVr, 3TC, ABC
**20**	6	6500	none	M184I (38%)	none	ABC	none	3TC	yes	none
**21**	9	520 000	K103N (84%)	K65R (98%) M184V (97%)	none	none	none	3TC, ABC, EFV, NVP	yes	3TC
9*	15	88 000	Y181C G190A	M184V	none	ABC	EFV	3TC, NVP	yes	none
**10**	6	69 000	K103N (98%) Y188C (16%)	none	none	none	none	EFV, NVP	no	none
**22**	6	7400	K103N (87%)	none	none	none	none	EFV, NVP	yes	none
**23**	15	6000	none	M184I (96%)	none	ABC	none	3TC	no	3TC
**11**	9	390 000	A98G (98%) K103N (82%)	M184V (98%)	none	ABC	none	3TC, EFV, NVP	no	none
**14**	12	50 000	V106M (72%) G190A (12%) F227L (11%)	none	none	none	EFV	NVP	no - LTFU	LPVr, 3TC, ABC
**24**	12	330 000	K103N (81%)	M184V (12%)	none	ABC	none	EFV, NVP ABC, 3TC	no - LTFU	none
**25**	18	15 000	V106M (91%) H221Y (98%)	M184V (98%)	none	ABC	EFV	3TC, NVP	no - LTFU	LPVr, 3TC, ABC

HIV genotype was determined using Next-Generation Sequencing methods of a selection of infants with plasma viral load >1000 RNA copies per mL, subject to sample availability. Mutation prevalence shown in brackets. *Indicates samples that only underwent Sanger consensus sequencing. Resistance phenotypes for the ART drugs commonly used in South African children were determined from the Online Stanford Database. Plasma ART levels were quantified using high performance liquid chromatography and were considered negative if at very low levels or below the level of quantification. Median infant age was 12 months, IQR 8–15 months; median viral load 24 000 HIV RNA copies per mL. No protease inhibitor (PI) resistance mutations were found for any of the participants. NNRTI: non-nucleoside reverse transcriptase inhibitor, NRTI: nucleoside reverse transcriptase inhibitor, ABC: abacavir, 3TC: lamivudine, NVP: nevirapine, EFV: efavirenz, LPVr: ritonavir boosted lopinavir, LTFU: loss to follow-up.

**Fig. 5:**
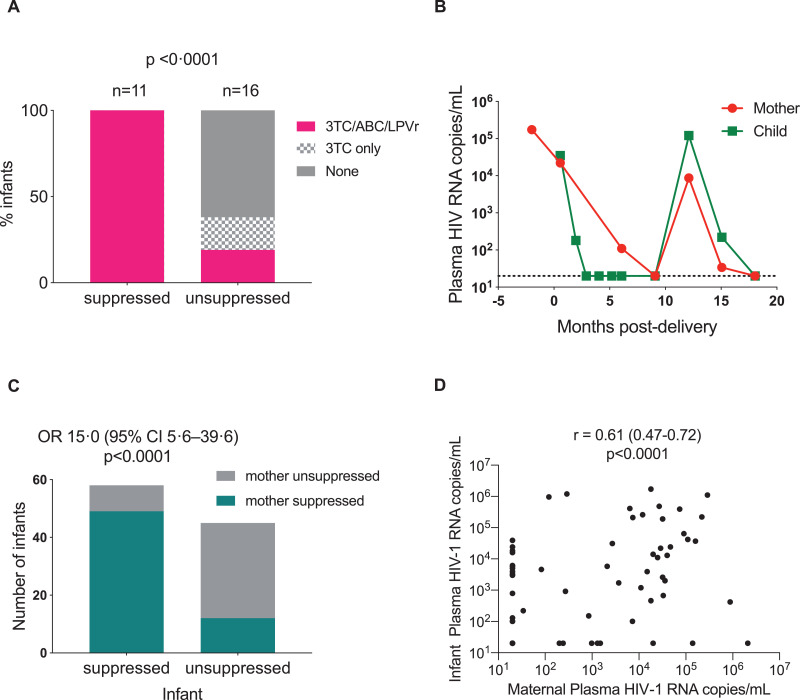
Determinants of infant plasma viraemia. (A) Results from plasma ART quantification from Table 3 are compared to those of 11 infants of similar age (median age 21 months) with plasma viral suppression (median duration of suppression 18 months) on cART. Negative levels includes very low levels and levels below the level of detection. P-value calculated using Fisher's exact test. 3TC: lamivudine, ABC: abacavir; LPVr: ritonavir boosted lopinavir. (B) Plasma viral load levels over time for a mother-child pair following combination antiretroviral therapy (cART) initiation, as an example of infant treatment success being determined by maternal cART adherence. Dashed line shows limit of detection (20 HIV RNA copies per mL). (C) A cross-sectional analysis of 101 mother-child pairs, >6 months on cART at the most recent timepoint of contemporaneous (within 14 days) plasma viral load measurement for the mother and child. Suppression is defined as a plasma viral load lower than the detectable limit (20 or 100 RNA copies per mL). Odds ratio is shown and p-value is calculated using Fisher's Exact test. (D) Spearman's non-parametric correlation of maternal and infant plasma load shown in (C).

To test further the hypothesis that children with virological failure were cART non-adherent, we compared plasma ART levels in children with virological failure (*n* = 16, Table 3) with children maintaining suppression of viraemia who by history were ART-adherent (*n* = 11). All 11 of the virally suppressed infants had all three prescribed ART drugs detectable at high levels in the plasma, while 13/16 (81%) of the infants with high viral loads had either no or only one ART drug detected at therapeutic levels (*p*<0.0001, Fig. 5A, Table 3). Together, these analyses indicate that the high frequency of failure to achieve suppression of viraemia on cART in the Ucwaningo Lwabantwana cohort of IU-infected infants is principally due to non-adherence.

Finally, in 101 children older than 6 months of age who had a plasma viral load measurement at the same time point as their mother, viral suppression in the mother and child was strongly associated (OR 15.0, 95%CI 5.6–39.6; *p*<0.0001, *r* = 0.61, 95%CI 0.47–0.72; *p*<0.0001) Fig 5B-D. Together, these data strongly support the conclusion that the same barriers to effective treatment affect both the mother and child, and if the needs of the mothers are addressed, then the challenges of administering ART to children are no longer a significant obstacle preventing ART adherence in the children.

## 5 Discussion

These studies were designed to explore the impact of early cART in IU-infected children and the opportunity to facilitate cure/remission. Initial plasma RNA and DNA viral loads in IU-infected infants were very low, with 12% of infants having undetectable viraemia at baseline, suggesting clear potential to ultimately achieve cure/remission in this group. The findings, however, are striking in highlighting the high-frequency failure of cART in this group, however early in life it is delivered. Barely one-third (37%) of cART-treated infants achieved suppression of viraemia by 6 months that was maintained beyond 12 months of age. Almost one quarter (24%) of IU-infected infants either died or were lost to follow-up by 6 months. In the subset tested, high-frequency cART failure did not result from drug-resistant mutations (DRMs). The barriers contributing to treatment failure are the same in both mother and child. This is an important result because it means that, if the needs of the mother can be successfully addressed, the practical challenges of administering cART to children, in most cases, will also be met. If durable suppression of viraemia in HIV-infected infants is to be achieved, and the undoubted opportunities to achieve cure/remission in HIV-infected children are to be fully realised, the unmet needs of transmitting mothers urgently need to be addressed first, whilst, at the same time, exploring options for long-acting or extended-release paediatric ART formulations. Finally, apparently superior treatment outcomes for the SoC tested group suggests that barriers to returning to clinic to collect test results selects out infants with inherently better outcomes, and that although PoC testing gives the opportunity to capture these vulnerable infants, more focus needs to put into supporting the maintenance of cART once initiated.

In South Africa, IU-infected infants usually receive cART transplacentally in addition to ART prophylaxis (AZT and NVP or NVP alone) immediately after birth (prior to HIV diagnosis). In this setting, neither this study nor another South African study by Technau et al. [6] showed superiority in initiating infant cART (AZT, NVP and 3TC) in the first 48 h of life following PoC testing and diagnosis, compared with cART initiation at a median of 10 days following SoC testing and diagnosis. Indeed, Technau et al. reported worse retention in care for the PoC tested infants, and here we observed that the SoC tested infants, who had returned to clinic from home for confirmatory testing, had higher rates of sustained viral suppression. Together, these findings suggest that HIV-infected infants successfully recalled from home following a positive birth test inherently may be more likely to succeed with treatment. However, the data to determine the proportion of infected infants successfully recalled were not available. It is possible that studies only analysing those infants successfully recalled may over-estimate positive treatment outcomes. The ability of PoC testing to initiate ART earlier in perinatally infected infants has been well-described in a range of African countries [20] and is demonstrated also here, but in countries such as South Africa where universal birth testing is already implemented, and where ART is reaching infected infants transplacentally and at birth via prophylaxis, the advantages of PoC over SoC testing may be reduced. Furthermore, these HIV-infected infants were born to mothers with a range of immune status and ART exposure, resulting in the wide heterogeneity of infant HIV DNA levels at baseline. It is possible that some of these infants were not in fact, early in infection and perhaps the relevance of initiating cART in the first hours of life has been overstated.

The high rates of treatment failure we present here, along with other studies describing the difficulties in treating paediatric HIV infection in sub-Saharan Africa [6,[21], [22], [23]], as well as in high-resourced settings [24,25] suggests a prevailing problem across the world. Perinatal HIV transmission, whether in high- or low-resourced settings, is concentrated in the most marginalised populations [26,27] who are often disengaged from conventional health care providers. Indeed, the Mississippi child is a case in point [9], whose remission only came to light following loss to follow up and ART non-adherence. Here we have found strong evidence showing cART failure in the child is due to cART non-adherence and is tightly linked to cART failure in the mother. The magnitude of relationship between mother and child suppression we found is much higher than has been recently described in older children [28], showing the critical role of the mother for consistent cART administration. On exploring the factors related to rate of infant viral rebound, counterintuitively, the strongest predictive factor was a longer duration of maternal cART in pregnancy. This could be explained by the mothers who were prescribed cART prior to pregnancy and defaulted treatment resulting in transmission *in utero*, being more likely to have difficulty administering infant cART compared to mothers who transmitted simply because they were diagnosed late in pregnancy. Interestingly, infants born at a younger gestational age and also those admitted to hospital as a neonate had lower rates of rebound, suggesting that additional support for the mother in the first weeks of life may be protective for virological control over the longer term. Older maternal age was also found to be protective against infant viral rebound. The importance of considering a child in the context of the mother is nowhere better illustrated than in the setting of perinatal HIV.

Infant mortality at 1 year was 9.3%, similar to other studies of South African IU HIV-infected infants [6] but high compared to national estimated infant mortality rate of 27–33/1000, albeit a figure only representing reported deaths, therefore a significant underestimate [29]. Our figure is also likely to be an underestimate, as the outcome for some of the infants lost to follow-up was unknown. The predictors of infant mortality were low birth weight, followed by neonatal hospital admission and baseline infant plasma viral load, the latter also reported by Technau et al. [6] Change of caregivers, home environment, comorbidities and other factors that were not systematically assessed by this study were likely playing pivotal roles. Moreover, the cause and circumstances of death were often not ascertained because most of the infants (9/15, 60%) died either at home or within a few hours of arrival at a hospital. This phenomenon in itself illustrates either the lack of access, poor engagement or mistrust in conventional health care providers. In-depth qualitative analysis of caregivers’ experiences may illuminate areas for improvement in provision of care for HIV-infected infants.

It is important to consider the potential limitations within this study. Most crucially, in our comparisons of outcomes following PoC testing versus SoC testing, it should be emphasised that this was not a randomised controlled trial. Indeed, there were some differences between the PoC and SoC groups that were unexpected, such as the proportion of females (74% vs 53%), and the proportion exclusively breast-fed (91% versus 68%), the clinical significance of which were unclear. With these differences at baseline, and the outcomes of the SoC tested infants who were not recruited onto the study unknown, a direct comparison of the PoC and SoC infants should be interpreted with caution. However, what is irrefutable, is that despite very early cART initiation, PoC tested infants’ outcomes are poor. It has been suggested that integrase strand inhibitor drugs can achieve suppression in early treated infants faster [25], which could mean that the poor viral suppression rates we describe will improve with the planned move away from paediatric regimens based on the notoriously unpalatable LPVr suspension. However, the strong association between viral suppression in the mother (taking one tablet once a day) with viral suppression in the child suggests that if the mother is cART-adherent, the cART regimen being utilised here in the children is effective and that unpalatability is not the over-riding factor causing failure. Although we found no evidence to suggest that drug resistance accounted for the high-frequency cART failure, our sample size was small, and we were only able to test children with a high plasma viral load, so it is possible that in some cases resistance contributed to cART failure. The direct testing of plasma showing a failure to detect cART in the majority of children with virological failure argues against this. Nevertheless, if these children continue to have viraemia and sub-optimal ART dosing, it is only a matter of time before ART resistance develops and contributes to treatment failure. Finally, our study was limited by the inability to determine the outcomes for the majority of infants who did not return to clinic and were lost to follow-up. Due to the poor coordination in HIV care in South Africa, it is possible that some of these infants relocated to another region to receive care without being formally being transferred, which may have led us to over-estimate true loss to follow-up.

ART has been widely successful in improving health, prolonging life and reducing HIV transmission events across sub-Saharan Africa. Furthermore, cART adherence in acutely infected women from a similar sociodemographic in KwaZulu-Natal with additional support can achieve excellent outcomes [30]. For the ∼0.6% of HIV-infected mothers in KwaZulu-Natal who transmit HIV IU [12] and who have already been failed by the PMTCT program, are unlikely to be successful with cART treatment for their child. In the absence of long-acting therapeutic interventions that can be implemented very soon after birth, unless these unmet needs are addressed, infant HIV-related morbidity and mortality will remain high.

## Author contributions

JRM coordinated the clinical aspects of the study, analysed the data and wrote the manuscript. NB, RF, KS, VN, NK, JR, KD, PK undertook the clinical management of the subjects, provided the clinical samples and contributed to writing the manuscript. VAV, MA, MM, AG, NG, PCM, TN, JA, KG, DB, TC, MB, NN, NI, EA, BDW, MCP and JMP undertook data analysis and helped in writing the manuscript. PG conceptualised and led the study, analysed the data and assisted in writing the manuscript. From the Ucwaningo Lwabantwana Consortium JvL, YG, KC, CK, RNB, MK and NM assisted with the identification of study subjects, provision of clinical samples for the study and writing of the manuscript, and RB, GM, and MdC undertook data analysis and helped in writing the manuscript.

## Declaration of Competing Interest

Dr. Martinez-Picado reports institutional grants and educational/consultancy fees outside the submitted work from AbiVax, Astra-Zeneca, Gilead Sciences, Grifols, Janssen, Merck and ViiV Healthcare. Dr. Millar reports personal fees from Cepheid, outside the submitted work.

## Appendix Supplementary materials

Image, application 1

Image, application 2

Image, application 3
