# Supplementary material for: HIGH-FREQUENCY failure of combination antiretroviral therapy in paediatric HIV infection is associated with unmet maternal needs causing maternal NON-ADHERENCE
Source: eClinicalMedicine. 2020 May 8;22:100344. doi: 10.1016/j.eclinm.2020.100344 (PMC7264978; doi:10.1016/j.eclinm.2020.100344)
Supplement: Supplementary file 1 [file mmc1.docx]

**SUPPLEMENTARY METHODS**

Where the confirmatory HIV PCR had a negative or indeterminate result, HIV-1 *gag* was amplified and sequenced using Sanger methods from both maternal and infant stored plasma^31^ and viral phylogenetic relatedness was used to confirm infant HIV-1 infection. In addition, positive HIV-1 DNA levels on two separate blood samples were also used to support the diagnosis.

Subject to sample availability, HIV from mother and infant plasma was reverse transcribed and amplified. Library preparation was performed using Nextera XT as per manufacturer guidelines and deep-sequencing was then performed using an Illumina MiSeq.^32^ Raw reads were blasted against Los Alamos Sequence Database (http://www.hiv.lanl.gov/) HIV-1 “Web Alignments 2017” to obtain the HXB2 alignment positions for each read. Reads covering possible drug resistance locations were translated to provide the amino acid percentages. Variants that were found in >10% of the sequence reads and drug resistance mutation locations had coverage of more than 30 were retained. Viral amino acid mutations were uploaded to the online Stanford University Genotypic Resistance Interpretation Algorithm V8·8 (http://hivdb.stanford.edu/) to obtain the drug resistance phenotype. All levels of resistance to antiretroviral drugs commonly used in South Africa were included. The presence of minor protease inhibitor (PI) resistance mutations in the absence of major PI mutations as defined by the Stanford database were disregarded.

Where sample was available (n=35), total proviral HIV-1 DNA levels were quantified using droplet digital PCR (BioRad, Hercules, California, USA) from PBMC as previously described.^33^ Samples were screened with two different primers/probe sets, annealing to the 5'*LTR*and *gag*conserved regions of HIV-1 genome.

***Supplementary Figure 1*: Infant outcomes at 24 months.**

40 infants were 24 months beyond enrolment and were grouped in accordance to their outcome at the 12 month analysis. The number from each group who achieved and maintained plasma viral suppression until 24 months of age are shown. CD8%; percentage of CD8+ T cells, CD4%; percentage of CD4+ T cells.

***Supplementary Figure 2*: Sensitivity analysis for infant plasma viral rebound.**

Kaplan-Meier analysis for all infants who achieved plasma viral suppression (plasma viral load lower than the detectable limit <20 or <100 copies per mL) and the time to viral rebound. A sensitivity analysis comparing the original definition of viral rebound (plasma viral load >1000 copies per mL on one occasion, or two measurements >100 copies per mL) to a more relaxed definition of one measurement >1000 copies per mL. P-value was calculated using the Log-Rank test.

***Supplementary Table 1*: Multivariate analysis of mortality, plasma viral suppression and rebound predictors.**

|  | **Mortality** | | **Time to Suppression** | | **Time to Rebound** | |
| --- | --- | --- | --- | --- | --- | --- |
| **Baseline variable** | ß coefficient | Multiplicative  Effect on Hazard Rate | ß coefficient | Multiplicative Effect on Hazard Rate | ß coefficient | Multiplicative  Effect on Hazard Rate |
| Group SOC |  |  |  |  | -0·443 | 0·642 |
| Study site |  |  |  |  |  |  |
| Infant birth weight (kg) | -0·566 | 0·568 |  |  |  |  |
| Infant sex male |  |  |  |  | -0·071 | 0·931 |
| Infant gestational age at birth (weeks) |  |  |  |  | 0·057 | 1·059 |
| Small for gestational age |  |  |  |  |  |  |
| Timing of maternal HIV infection  - chronic vertical |  |  |  |  |  |  |
| Days of maternal ART in pregnancy |  |  |  |  | 0·002 | 1·002 |
| Self-reported maternal ART non-adherence in pregnancy |  |  |  |  |  |  |
| Maternal age (years) |  |  |  |  | -0·027 | 0·973 |
| Infant prophylaxis |  |  |  |  |  |  |
| Age infant ART initiation |  |  |  |  |  |  |
| Infant CD4 count (cells/uL) |  |  |  |  |  |  |
| Infant CD4% |  |  | 0·008 | 1·008 |  |  |
| Infant CD8 count (cells/uL) |  |  | 0·007 | 1·007 |  |  |
| Infant CD8% |  |  |  |  |  |  |
| Infant CD4:CD8 |  |  |  |  |  |  |
| Infant log_10_ HIV RNA copies/mL | 0·062 | 1·064 | -0·360 | 0·700 |  |  |
| Maternal CD4 count (cell/uL) |  |  |  |  |  |  |
| Maternal CD8 count (cells/uL) |  |  |  |  |  |  |
| Maternal CD4:CD8 |  |  |  |  |  |  |
| Maternal log_10_ HIV RNA copies/mL |  |  |  |  |  |  |
| Infant feeding |  |  |  |  |  |  |
| Neonatal hospital admission | 0·529 | 1·700 |  |  | -0·206 | 0·814 |

A classical Cox model was used with a LASSO penalty to determine the effect of 25 variables at baseline on the rate of the following outcomes; mortality, plasma viral suppression and plasma viral rebound. The relevant covariates selected by the model are shown, with blank cells representing the irrelevant covariates eliminated via the LASSO. The ß-coefficient gives the influence of the variable in the units stated, whereas the “multiplicative effect on hazard rate” (Exp(ß)) gives the factor by which the rate to the outcome is affected by the variable, analogous to a hazard ratio. Variables are highlighted, green for decreasing the rate of the outcome, orange for increasing it.

[31] Adland E, PaioniP, Thobakgale C, et al. Discordant ipact of HLA on viral replicative capacity and disease progression in paediatric and adult HIV infection. PLoS Pathog 2015; 11:e1004954.

[32] Bonsall D., GolubchikT., de CesareM., et al. A comprehensive genomics solution for HIVsurveillance and clinical monitoring in a global health setting .2018. <https://www.biorxiv.org/content/10.1101/397083v4> (accessed August 23, 2019).

[33] Morón-López S, Puertas MC, Gálvez C, et al. Sensitive quantification of the HIV-1 reservoir in gut-associated lymphoid tissue. PLoS One 2017;12:e0175899.
