# Supplementary material for: HIGH-FREQUENCY failure of combination antiretroviral therapy in paediatric HIV infection is associated with unmet maternal needs causing maternal NON-ADHERENCE
Source: eClinicalMedicine. 2020 May 8;22:100344. doi: 10.1016/j.eclinm.2020.100344 (PMC7264978; doi:10.1016/j.eclinm.2020.100344)
Supplement: Supplementary file 3 [file mmc3.pdf]

PROTOCOL NUMBER:

....
For office use only

[Grab your reader's attention with a great quote from the document or use this space to emphasize a key point. To place this text box anywhere on the page, just drag it.]

RESEARCH OFFICE CONTACT DETAILS: Biomedical Research Ethics Administration, Westville Campus, Govan Mbeki Building, Private Bag X 54001, Durban, 4000, KwaZulu-Natal, South Africa; Tel: +27 31 2602486; Fax: +27 31 2604609; Email: BREC@ukzn.ac.za ;
Website: http://research.ukzn.ac.za/Research-Ethics/Biomedical-Research-Ethics.aspx

BIOMEDICAL RESEARCH ETHICS COMMITTEE APPLICATION FORM[1]

Application to the UKZN Research Ethics Committee for ethics review of new research projects
(For research on human participants)

SECTION A:

APPLICANT/PRINCIPAL INVESTIGATOR:								* For UKZN statistical reporting purposes			
Title: Mr		Ms		Mrs		Dr		Prof	X	(Select option)	
Name : Philip Goulder											
*Gender: Male											
*Race: White											
UKZN College: College of Health Sciences											
UKZN School/Discipline:	HIV Pathogenesis Programme									NA	
Hospital/Institution where employed:	University of Oxford, UK									NA	
Professional status:	Professor of Immunology										
Postal address:	The Peter Medawar Building for Pathogen Research, South Parks Rd, Oxford OX1 3SY, UK										
Contact phone Numbers: Office: +44 1865 281884											
Mobile number:	+44 7595 721053										
Fax number:	+44 1865 281236										
Email address:	Philip.goulder@paediatrics.ox.ac.uk										
Full/Part time Employment: Full time											
Current HPCSA Number (or equivalent): N/A<br>*if registration is pending, submit proof of application											

Purpose of research: If postgraduate degree *(Please tick)* [N/A: Research not for degree purposes]	Hons	MMedSc	MMed	MSc	MFamMed	MHIV	PhD

Other degree not listed above:			
Student Number and year of study: (*if applicable*)			
If for postgraduate degree, please confirm whether the application has been reviewed and approved by your school's Academic Leader (Research):	Yes		No

[1] Note: This application must be self-sufficient. Sections marked "see protocol" are unacceptable and will be returned to the applicant.

If yes, provide approval date and attach approval letter:
Name and qualifications of Supervisor
Name and qualifications of Co-supervisor
If not for degree purposes, state other (example, self-initiated research): Self-initiated research

Has this study been, or is it likely to be, submitted to any other Research Ethics Committee?	Yes		No<br>X		N/A	

If yes, please name the Committee/s and or institution and give outcome - i.e. approved/rejected/pending/not applicable? *(If approved, attach approval letter)*	
**Please state number of Co-investigators in project:[2]**<br>**(if additional space is required for more investigators details please add to the end of application)**	
**CO-INVESTIGATOR/S ROLE IN PROJECT**	** For UKZN statistical reporting purposes*
Name: **Professor Thumbi Ndung'u**	
Faculty: College of Health Sciences	
Department: HIV Pathogenesis Programme	
*Gender: Male	
*Race: Black	
Role: Co-investigator	
Signature of Co-Investigator:	
Name: **Dr Zodumo Mvo**	
Faculty:	
Department: UMKHUSELI INNOVATION AND RESEARCH MANAGEMENT (RF) NPC	
*Gender: Female	
*Race: Black	
Role: Study coordinator	
Signature of Co-Investigator:	
Name: **Dr Nomonde Bengu**	
Faculty:	
Department: Lower Umfolozi War Memorial Regional Hospital, Empangeni, KwaZulu-Natal	
*Gender: Female	
*Race: Black	
Role: Study co ordinator	
Signature of Co-Investigator:	
Name: **Kenneth John Sprenger**	

[2] Please note that because of conflict of roles and interests that can arise, academic supervisors and co-investigators should be separate individuals.

Faculty: Paediatrics (OPD), Stanger Hospital
Department: Paediatrics (OPD), Stanger Hospital
*Gender: Male
*Race: White
Role: Study co ordinator
Signature of Co-Investigator:
Name: **Vuyokazi Ntlantsana**
Faculty: College of Health Sciences, University of KwaZulu-Natal
Department: Obstetrics and Gynaecology
*Gender: Female
*Race: African
Role: Study co ordinator
Signature of Co-Investigator:
Name: **Dr Kogielambal Chinniah**
Faculty: Paediatrics
Department: Mahatma Gandhi Memorial Hospital, Phoenix
*Gender: Female
*Race: Indian
Role: Study coordinator
Signature of Co-Investigator:
Name: **Dr Jane Millar**
Faculty: UMKHUSELI INNOVATION AND RESEARCH MANAGEMENT (RF) NPC
Department: UMKHUSELI INNOVATION AND RESEARCH MANAGEMENT (RF) NPC
*Gender: Female
*Race: White
Role: Study coordinator and co-investigator
Signature of Co-Investigator:
Name: **Dr Rowena Fillis**
Faculty: UMKHUSELI INNOVATION AND RESEARCH MANAGEMENT (RF) NPC
Department: UMKHUSELI INNOVATION AND RESEARCH MANAGEMENT (RF) NPC
*Gender: Female
*Race: Coloured
Role: Study coordinator

Signature of Co-Investigator:				
Has the Principal Investigator or any of the co-investigators been previously/or are presently being investigated for alleged research misconduct?<br>*(If yes, please provide details and dates)*	Yes		No<br>X	

**FUNDING OF THE RESEARCH:**				
Has funding been secured?	Yes<br>X		No	
Amount: R 16,912,000				
Name of funder: *(full details)* Wellcome Trust (Principal Investigator: Goulder), duration 5 years (2015-2020)				
Is this project funded from a US DHHS funding source?	Yes		No<br>X	
If yes, name the federal funding agency:				
Can this project proceed without funding?<br>*(give a brief explanation)*	Yes		No<br>X	
Has an application for funds been made to other sources to support this project?	Yes		No<br><br>X	
If yes, state name/s of funding agency and amount requested: N/A				

Note:

For all US Federally funded studies (e.g. NIH, CDC, NIAID, DAIDS, NIMH, etc), one complete copy of the original funding application and approval must accompany the BREC ethics application.

All University contracts need to be uploaded on the Contracts Management online submission form with either the signed **Approval letter** (non-research) **or Form 1**(research related). The website link to the system is http://legalservices.ukzn.ac.za/ContractsManagement.aspx

If you require assistance with the completion of the online submission form, or with any aspect of the new system, please contact Mr Rendra Phalad on Ext 7455 for all contracts (non-research contracts), and Mr Deon Moodley on Ext 8199 (for research contracts).

FAILURE TO MAKE FULL FINANCIAL DISCLOSURES WILL DELAY ETHICS APPROVAL

Please indicate whether a BREC review fee is applicable for this study?<br>(See Fee Schedule on BREC Website)	Yes	X	No	
If Yes, is the study covered by your Centre/Unit's annual levy fee to BREC?	Yes		No	X

**TYPE OF RESEARCH** *(please tick)*				
Expedited		Full review	√	

Note:

* Expedited review only applies to minimal risk studies – e.g. retrospective chart reviews, studies on stored samples etc., for details see BREC ToR and SoP at *http://research.ukzn.ac.za/Research-Ethics/Biomedical-Research-Ethics.aspx*

SECTION B:

NATURE OF STUDY

Quantitative

Type of Study: *(please tick)*	Epidemiological	Observational clinical study	Experimental	Clinical Trial	Observational √
	Retrospective Chart Review	Prospective Chart Review	Laboratory study on stored samples	Other:(Specify)	

Qualitative

1. THE PROTOCOL FOR STUDY

1.1 Full title of research project: *(Please DO NOT use abbreviations or acronyms)*

Impact of early diagnosis and anti-retroviral therapy (ART) initiation at birth in children with *in utero* HIV infection

1.2 Aims (what you hope to achieve) and objectives (how you will achieve your aims) of study:

Aims:

i) To demonstrate the feasibility of early diagnosis and ART initiation within the first 48hrs of life in children with in utero HIV infection;

ii) To determine the impact of ART initiation within the first 48hrs of life on viral burden (viral load, viral reservoirs in the peripheral blood) and on anti-HIV immune responses through the first 2-5 years of life.

Objectives:

We will test children we define as 'at risk' (defined below) for HIV infection on the day of birth, and will ensure that ART is initiated within 48hrs of birth. We here define 'at risk' as babies either born to HIV-infected mothers who have not received any ART during pregnancy; or only started ART less than 4 weeks before delivery; or who have been non-adherent to ARVs during pregnancy; or who first tested positive for HIV infection during labour; or who seroconverted during pregnancy.

Study Plan:

Study sites: The project will be initiated at Stanger Hospital, and we plan to include Edendale Hospital, and possibly also Imbalenthle Clinic, as additional sites once the study has been initiated. The Lower Umfolozi War Memorial Regional Hospital (LUWMRH) in Empangeni, KwaZulu-Natal was added as an additional study site in 2016. The following clinics will serve as *referral* sites for LUWMRH: Buchanana Clinic, Khandisa Clinic, Isiboniso Clinic, Ngwelezane Clinic, Mkhontokayise Clinic, Phaphamani Clinic, Thokozani Clinic, Mandalanzani Clinic, Sokhulu Clinic, and Umbonanmbi Clinic. Participants will not be enrolled or studied at these referral sites. The Mahatma Gandhi Memorial Hospital in Phoenix, KwaZulu-Natal, was added as an additional study site in 2017.

Identification of 'at risk' infants: Infants defined as 'at risk', using the criteria described above, will be identified by the research nurses/counselors employed on this research study.

Anti-retroviral therapy (ART):

The child will receive nevirapine (NVP) alone or NVP and zidovudine (AZT) dual prophylaxis as per South African national guidelines as soon as possible after birth and will be given by the Department of Health maternity staff. Informed consent will be sought from the mother in order for a blood sample to be taken from the child in order for viral load testing at Global Laboratory or qualitative HIV nucleic acid point of care testing via the Cepheid GeneXpert machine if available. This will allow test results to be available and ART initiation within 48hrs of birth in all newborns diagnosed as HIV-positive. ART will be initiated as per South African guidelines with the combination of AZT, lamivudine (3TC) and NVP used in newborns then switched

to abacavir (ABC), 3TC and ritonavir boosted lopinavir (Kaletra) at the earliest 42 weeks gestational age as per the National guidelines as per the National guidelines.
Schedule of follow up and blood sampling: The schedule of blood sampling and follow up for HIV positive children enrolled onto the study is shown below in Fig 1. Blood samples will be taken to monitor viral load and CD4 count and also for studies of viral reservoir and anti-HIV immune responses (detailed below). Routine baseline and follow up tests will be taken including, haemoglobin, transaminases and lipid profile in keeping with the South African National ART guidelines. If the mother consents, we will also take blood from the mother at enrollment, then at 1 month, and 3 months post-natally, and then 3 monthly at the same visits to the clinic shown in Fig 1. The reason for this is that mothers who have transmitted HIV to the child are in most cases either recent seroconverters in pregnancy, and hence new to ART, or ART non-adherent. It is vitally important both for the baby and the mother that the mother is optimally adherent post-natally - this study can help to determine whether there are problems with maternal ART adherence by more frequent monitoring than would be possible outside of research study settings. In cases where the mother of an HIV-infected infant had themselves been infected with HIV perinatally, and if the grandmother provides informed consent, we would also request a one-off 50mL blood sample from the enrolled child's maternal grandmother in order to confirm virus transmission from grandmother to mother.

Those mothers who had an acute HIV infection during pregnancy (i.e. a negative test later followed by a positive test) and whose babies test negative at birth will also be asked to consent for a once off 50mL blood sample.

As control groups if mothers provide informed consent, we will also take a one-off blood sample of 2.5mL from a group of 'at risk' infants aged <48hrs who tested HIV negative (HIV exposed uninfected – HEUs) as well as from a group of HIV-unexposed uninfected infants (HUUs).These control babies will not be followed up longitudinally.

An additional comparison group will be provided by uninfected or infected siblings of the infected babies. These siblings will have a weight-based blood sample taken and followed 6 monthly for 1 year then annually.

Collateral information with regard to HIV acquisition will be obtained from the total number and gender of deliveries at each site from the maternity ward delivery book, as well as the National Health Laboratory birth HIV PCR results for the district the study site is located in (Umhlathuze, eThekwini, iLembe and Umgungundlovu).

Fig 1. Planned blood sampling times and volumes in IU HIV-infected infants. Blood samples will be taken at the ages shown in IU infected infants. Sample volumes allowable for research bloods are calculated using the Stellenbosch University Health Research Ethics Committee Guidelines. *Weight for babies uses WHO growth charts, blood volumes will be adjusted according to weight as per guidelines. Research blood volumes allowed are calculated at 0.8ml/kg/bleed using the weights shown, with a maximum limit of 2.4ml/kg per 28 days. Anticipated lymphocyte and CD4 T-cell yield from the sample volume shown from Shearer *et al JACI*, 2003. A blood sample will also be sought from the mother on d1 in order to confirm authenticity of transmission.

Age	d0	d1	1m	2m	3m	4m	5m	6m	9m	12m	15m	18m	21m	24m	27m	30m
Weight*	3-3.5kg					6kg				10kg				12-13kg		
Sample volume allowed/bleed	2.6ml					4.8ml				8ml				10ml		
Lymphocytes/ml	$4\text{-}6\times10^6$/ml										$3\text{-}4\times10^6$/ml					
Lymphs yield	$12\text{-}14\times10^6$					25×10^6				34×10^6				$30\text{-}40\times10^6$		
CD4 T-cell yield	$6\text{-}7\times10^6$					12×10^6				16×10^6				$12\text{-}16\times10^6$		
Blood sampling	0.5ml	2.6ml	2.6ml	3ml	4ml	5ml	5ml	6ml	7ml	8ml	8ml	9ml	9ml	10ml	10ml	10ml

Clinical research team: The clinical research team will comprise between one and two Research Nurse/Counselors at each clinical site, depending on how busy is that site, and a Research Coordinator who will oversee the running of the study and who will liaise between the clinic staff at the clinical sites and the laboratory personnel at Global and at the Doris Duke Medical research Centre, University of KwaZulu-Natal. All of the research team will be funded from the funding that has been secured to support this research.
Laboratory studies planned: In addition to the viral loads and CD4 counts being undertaken at Global Laboratories in Durban, studies will also be undertaken at the HIV Pathogenesis Programme within the Doris Duke Medical Research Institute to determine the size and localisation of the viral reservoirs in peripheral blood of infected infants enrolled, and to undertake studies of the immune activity present in these children. Screening for cytomegalovirus (CMV) will be undertaken to account for CMV infection as a potential confounder causing immune activation.
Location of laboratory studies planned: Aside from the viral load and CD4 testing that will be done in Global Laboratories, Durban, most of these assays described above will be undertaken under the supervision of Prof Thumbi Ndung'u in the HPP labs in Durban. This work will be in collaboration with Prof Sharon Lewin at the University of Melbourne, Australia. A subset of the immunology work will be done in the laboratories of Prof Goulder in Oxford. It is planned therefore that the laboratory work will be undertaken within the HPP in Durban, and a subset will be undertaken overseas under the supervision of Prof Goulder in Oxford, UK and other overseas laboratories including Prof Lewin in Melbourne, Australia.

1.3 Hypothesis to be tested, or Research Question to be answered:

Hypotheses to be tested:
In spite of highly effective prevention of mother-to-child transmission programmes, a small percentage of children born to HIV-infected mothers are infected at birth.
Hypothesis 1: We hypothesise that we will be able to identify 150-200 in utero infected children over a 3-year period and initiate ART within 48hrs of birth.
Hypothesis 2: We hypothesise that we will be able to maintain high follow up rates and high adherence to

ART so that, by 24 months of age, 35-50% of enrolled children will have sero-reverted (lost HIV antibody reactivity by Western blot testing), suggesting that ART initiated this early in life may have the effect of reducing viral reservoirs to very low levels.
Hypothesis 3: We hypothesise that anti-viral immune responses are detectable in the first 48hours of life in 'at risk' HIV-exposed uninfected infants, and that these differ when compared to HIV-unexposed infants and also when compared to in utero HIV-infected infants. Similarly, we hypothesise that anti-viral immune responses may be detectable in HIV-exposed uninfected siblings of HIV-infected infants.

During the first 2 years of this study we have found a 2.5:1 ratio of females to males in our cohort of in-utero infected infants. We now would like to further explore this difference in acquisition and/or survival of in-utero HIV infection.
Hypothesis 4: We hypothesise that the ratio of male to female stillbirths is higher in HIV positive mothers than HIV negative.
Hypothesis 5: We hypothesise that there are significantly more females with a positive birth HIV PCR than males.

1.4 Summary of the proposed research (restrict to 100 words)

There is increasing evidence that early ART is effective in reducing the size of the HIV reservoir. In particular this is the case in HIV-infected children. The recent case of the Mississippi child who received ART at 30hrs of life supported this hypothesis. This child maintained undetectable levels of HIV for 27 months after stopping ART, until rebounding aged almost 4yrs. The current studies are designed, first, to determine whether in utero babies can be identified and treated with ART within 48hrs in significant numbers in South Africa; and, second, whether ART initiation at birth does indeed allow viral reservoirs to be reduced to very low levels.

1.5 Keywords (for database):

HIV, HIV Cure, Viral reservoirs, antiviral immunity

1.6 Background and Literature Review (maximum 1 page):

The dramatic success of programmes that have been established to prevent mother-to-child transmission of HIV have reduced transmission rates to around 1.5-2%[1]. However there remains this small fraction of children born to HIV-infected mothers who slip through the net and become infected. With approximately 300,000 babies born to HIV-infected mothers in South Africa each year, this amounts to up to 6,000 infected babies each year. Thus there is a drive towards early diagnosis of HIV infection in newborns. The first aim of this study therefore is to determione the feasibility of the proposed approach of HIV testing on the day of birth of all 'at risk' infants.

The second aim of the study relates to the HIV cure research field. There is increasing evidence that the size of the latent viral reservoir that HIV established in resting memory CD4 T cells is limited in size the earlier that ART is initiated[2-4]. This is the case both in adult infection but espeically so in paediatric infection where there is a low frequency of central memory CD4 T cells which where the HIV reservoir is typically localised. In children receiving ART early, viral reservoir decays substantially more rapidly than in older children and chronically infected adults. In addition, the failure to detect HIV in the Mississippi child[5] (who received ART at 30hrs' age) for 27 months after the child had stopped ART, in spite of the most sensitive assays of HIV reservoirs measurement being used, was further evidence that early ART can substantially reduce viral reservoirs especially in children.

The Mississippi case raises the possibility that cure is achievable in paediatric infection if ART is initiated immediately. The significance of cure is clear, but caution is urged in extrapolating from a single case. This case may be too exceptional to be reproducible. Its reproducibility is critical, first, to verify the finding; and,

second, to define the mechanisms underlying it. Development of a better understanding of of the Mississippi case needs sufficient numbers of infected babies receiving early ART to perform the study.

One of the challenges to this study is to be able to determine what differences there are between in utero HIV-infected infants who have the appearance of cure or remission, as in the case of the Mississippi child, and HIV-uninfected infants. To determine these differences we will study three control groups of infants: - - -

- First, infants who are born to HIV-positive mothers, but who are HIV-uninfected: these are termed HIV-exposed uninfected (HEU);
- Second, HIV-uninfected infants born to HIV-uninfected mothers: these are termed HIV-unexposed uninfected (HUU). There is a wealth of accumulating data indicating that the HEU group are immunologically distinct from the HUU group of infants[6].
- Third, HIV-uninfected or infected siblings of in-utero HIV infected infants . The exposed-uninfected siblings are like the first group of controls but they have been exposed to the same maternal virus as the infected infant. While the HIV infected siblings allow comparison of HIV immune control in genetically similar children (25% of siblings are HLA identical and 50% of siblings share half HLA alleles) who have been infected with a very similar virus from the same mother.

The prevailing view is that intervention in addition to early ART would be needed to achieve HIV cure in such cases. Currently there are several options available such as T-cell vaccines or infusion of broadly neutralising antibodies such as PGT121[7]. These are potential options in the future, should their development prove sufficiently promising. However, the purpose of this current study is to determine whether early ART can be achieved successfully in a substantial number of HIV-infected infants, and whether this early initiation of ART would result in substantially lowered viral reservoirs over the course of time in the infants enrolled.

References:

1. Moodley P, Parboosing R and Moodley D. Reduction in perinatal HIV infections in KwaZulu-Natal, South Africa, in the era of more effective prevention of mother to child HIV transmission interventions (2004-2012). *JAIDS*, 2013, 63: 124-33.
2. Li JZ, and Gandhi RT. The Sooner, the Better: More Evidence that Early Antiretroviral Therapy Lowers Viral Reservoirs in HIV-Infected Infants. *J Infec Dis*, 2014, May 21. pii: jiu298. [Epub ahead of print]
3. Luzuriaga K, et al. HIV proviral reservoirs decay continuously under sustained virologic control in early treated HIV-1-infected children. *J Infec Dis*, 2014, May 21. pii: jiu297 Epub ahead of print
4. Persaud D, *et al.* Dynamics of the resting CD4 latent reservoir in infants initiating HAART at less than 6 months of age. *AIDS*, 2012. 12: 1483-90.
5. Persaud D, *et al*. Absence of detectable HIV-1 viremia after treatment cessation in an infant. *New Eng J Med*, 2013, 369: 1828-35.
6. Slogrove AL *et al*. A Prospective Cohort Study of Common Childhood Infections in South African HIV-exposed Uninfected and HIV-unexposed Infants. *Pediatr Infect Dis J*. 2017 Feb;36(2):e38-e44.
7. Barouch D, *et al*. Therapeutic efficiency of potent neutralizing HIV-1 specific monoclonal antibodies in SHIV-infected rhesus monkeys. *Nature*, 2012, 503: 224-8.

2. PLAN OF INVESTIGATION FOR STUDY

* In the case of Higher Degrees, please state name and School of person consulted regarding the design:

N/A

2.1	Is this a retrospective chart review with no human contact?	Yes		No	X
2.2	Is this a study of stored tissue?	Yes		No	X

2.3 Are host genetic factors being studied?	Yes	X	No	
2.4 How many hours per week will the PI devote to this project?<br>*(Timetable the project in terms of the resources and time available)*<br>Approximately 10hrs/week for the 5yr funding period				

**3. STATISTICAL PLANNING AND DATA ANALYSIS**				
3.1 Has this project been approved by a professional statistician?<br>If No, please justify.	Yes	X	No	
3.2 If answered "yes" to (3.1), provide the name of the statistician: Jacob Hurst				

3.4 Please provide a brief overview of statistical and data analytic considerations, including:
How was the number of participants determined? Please include assumptions made in any power analysis (e.g. control incidence or mean and standard deviation of primary outcome variable, desired or anticipated effect of treatment or intervention, level of statistical significance and desired power), and list all planned statistical methods to be used. For descriptive studies list statistical operations to be performed.

These studies seek:
1. To determine the feasibility of identifying and initiating ART within 48hrs of birth in newborn infants in South Africa; and
2. To determine whether ART initiated at this early stage will result in sufficient lowering of viral reservoirs such that the reservoir will become undetectable using the most sensitive assays available, and anti-HIV immune responses, include Western blot reactivity, will all be negative within 24 months of treatment. This is the primary endpoint of this second aim of the study. For the purposes of this study we have defined such a child as a 'functional cure'. Power analysis indicates that recruitment of 100 children will give a 90% chance of observing a least one 'cure' if the cure rate per enrolled child is as low as 2.3%. This assumes 20% loss-to-follow-up, 10% ART failure and 5% mortality (that is, if 65 of the enrolled 100 children have maintained viral suppression on ART, and have not been lost to follow up or have died). Secondary endpoint analyses to determine if baseline CD4 cell counts/HIV DNA metrics are associated with the primary endpoint are highly dependent on the cure rate. Assuming a cure rate of 10-25%, there is an 80-97% chance that baseline CD4 cell percentage would show a statistically significant difference ($p<0.05$) if the difference in CD4% between the two groups (ie Group A: children who are functionally cured by the definition above; Group B: children who are not functionally cured, by the definition above) is 10% (mean CD4% 50% within the 'cure' group and 40% in the 'non-cure'). These power calculations were carried out using one-tailed unpaired t-tests, type-I error 5%, within group SD 10%.
3. To compare the immune profiles of in-utero HIV infected infants aged <48hrs to 'at risk' HIV exposed but uninfected infants aged <48hrs who were at similar risk of in-utero infection and then to HIV unexposed uninfected infants aged <48hrs.
4. To further explore our preliminary finding of a higher rate of in-utero HIV infection in females than males by looking at the rate of stillbirths, live births and HIV positive birth PCRs in HIV exposed and unexposed male and female babies at our study sites. This sex difference may reveal valuable a valuable insight into HIV susceptibility and progression.
5. To compare the immunological and genetic profiles of IU HIV infected infants to their uninfected or infected siblings in order to investigate factors protecting against perinatal HIV infection or controlling the virus once infected.
6. To determine virus or host immune factors contributing to in-utero HIV transmission after acute HIV infection in pregnancy.

3.5 For *qualitative* studies: What is the analytic paradigm to be used for analysis of the data?

N/A

4. PARTICIPANTS IN THE STUDY

4.1 Is this a multi-national study? (*If yes, state collaborating countries)*	Yes			No	X	

4.2 List all sites in South Africa in which the project will be carried i.e. geographic location (e.g. KwaZulu-Natal) and type of place (e.g. hospital, clinic, schools, community etc).

Edendale Hospital and Imbalenthle clinic, Kwa-Zulu Natal;

Stanger Hospital, KwaZulu-Natal;

Lower Umfolozi War Memorial Regional Hospital, Empangeni

Mahatma Gandhi Memorial Hospital, Phoenix, KwaZulu-Natal

4.3 Source: *(Please indicate number per group)*	Inpatients	Outpatients	Volunteers	Animals
			300 - 400	

4.4 Age (human studies) *(Please indicate number per group)*	Neonates (<28 days)	Infants (1-11 month)	Children (1-12 years)	Adolescent (13-17 years)	Adults
	250 - 300				150 - 300

4.5 Is there a control group(s)?	Yes	X	No		

4.6 Demographic profile of participants *(please tick ALL appropriate boxes below.)*

4.6.1 Gender: Female [X] Male [X]

4.6.2 Population Group: Black [X] Coloured [x] Indian [x] White [x]

4.6.3 Language Group/s: Specify……… N / A……

4.7 Describe the recruitment process in detail for all groups.

The research nurse/counselor will identify 'at risk' infants either when the mother presents to labour ward. We here define 'at risk' as babies either born to HIV-infected mothers who have not received any ART; or only started ART less than 4 weeks before delivery; or who have been non-adherent to ARVs during pregnancy; or who first tested positive for HIV infection during labour; or who seroconverted during pregnancy. The Research Nurse/Counselor will seek informed consent from the mother/caregiver shortly after delivery of the child, or before if appropriate, to enroll the mother and the baby onto the study. Once the mother has provided informed consent, a blood sample will be taken from the child and either an HIV viral load or point of care test via the GeneXpert qualitative HIV kit if available to determine HIV diagnosis. The result will be available within 24 hours for a viral load and 90 minutes for a GeneXpert test. If the child is testing HIV-negative it will be disenrolled from the study and follow up care will be taken over by the clinic according to current standard of care. However, before disenrollment, consent will be sought from the mother to include the infant on the study as an 'at risk' HIV-exposed uninfected control infant. If informed consent is obtained, a one-off 2.5mL blood sample will be taken from the infant to provide an HIV exposed but negative control sample. If the child is testing HIV-positive from this initial sample, a second sample will be taken to confirm the diagnosis, and ART will be commenced. At the same time a sample from the mother/father will be taken to confirm the authenticity of the maternal transmission of HIV to the child.

The two HIV positive control arms of the study, Arms 2 and 3 comprise *in utero* HIV-infected children who have been diagnosed HIV-infected and anti-retroviral therapy (ART) initiated between day 3 and day 14; and between day 15 and day 21 of life, respectively, but not within the first 48 hours of life. These infants will have been identified typically as a result of dried blood spot testing (DST) which is standard of care. When the mother and child return to the clinic for this initial visit the research nurse/counselor will seek informed consent from the mother/father to enroll the mother/father and the baby onto the study in either Arm 2 or 3 depending on the current age of the baby. Follow up of the children

enrolled in the HIV positive control groups will be exactly as described for the study group.

The HIV negative control groups will comprise of an unrelated group and a related group. The unrelated group will consist of HIV exposed uninfected (HEU) infants <48hrs old and a group of HIV unexposed uninfected (HUU) infants <48hrs old. Following the mother's consent, these infants will have a once off 2.5mL blood sample taken then they all will be disenrolled from the study. The related group will consist of uninfected or infected siblings, who will have a weight based (0.8mL/kg maximum) blood sample taken at enrollment, 6 months, 1 year then annually.

Consent for obtaining cord blood will be requested from mothers who are having planned caesarian sections for whatever indication. Following the mother's consent, once the placenta has been delivered and discarded, the cord blood will be drawn into a blood bag containing anti-coagulant.

Those mothers who had an acute HIV infection during pregnancy (i.e. a negative test later followed by a positive test) and whose babies test negative at birth will also be asked to consent for a once off 50mL blood sample before they are discharged from hospital after delivering and then will be disenrolled from the study.

Supplementary information regarding the sex ratios of HIV exposed still births, births and HIV transmission will be collected from the hospital delivery record book at each site and the National Health Laboratory birth HIV PCR results for each study site district during the time of our recruitment.

4.8 Will incentives be offered to facilitate recruitment?<br>*(If yes, describe in detail)*<br><br>No incentives will be offered at recruitment, however reimbursements will be offered at each follow up clinic (see immediately below).	Yes		No	X	
4.9 Will participants be reimbursed in some way for participation?<br>*(If yes, describe in detail) See SA DoH Guidelines on BREC Website*<br><br>Participants will receive R175 at each visit to the outpatient department as per DoH guidelines in order to cover the cost of travel and some food.<br>Additional compensation will be considered for participants based on the inconvenience, expenses and time spent in the context of their participation in our study, e.g. for participants with long travel to the study site.	Yes	X	No		
4.10 Will reimbursement for participants and investigators be in accordance with:<br>*(If no, please explain)*<br>• Guidelines for Good Practice in the Conduct of Clinical Trials in Human Participants in South Africa: Department of Health (2006) and;<br>• Ethics in Health Research: Principles, Structures and Processes: (2004)?<br>• Current SA DoH Guidance on reimbursement (*See BREC website*)	Yes	X	No		
4.11 Will participants be insured against research related injury?<br>*(If yes, please provide details; If no, please provide rationale)*<br>*Mandatory for Clinical Trials*<br>This is not applicable as their participation will be purely in accordance with South African treatment guidelines.	Yes		No	X	

4.12 List in detail the inclusion and exclusion criteria.

Inclusion criteria:

1. Newborn infants, including singletons and multiple births, aged <48hrs born to mothers who:
a) Did not receive ART during pregnancy; or who
b) Received ART <4 weeks prior to delivery; or who
c) First tested HIV+ve during labour; or who
d) Were non-adherent to ARVs during pregnancy; or who
e) Seroconverted during pregnancy (i.e. had a negative HIV test at booking into the antenatal clinic but subsequently tested positive).
OR Infants testing HIV-positive within the first 21 days of life but in whom ART was not initiated within the first 48hrs of life
OR HIV-uninfected infants born to an HIV-positive mother who tested HIV negative, enrolled within 48hrs of life
OR HIV-uninfected infants born to an HIV-negative mother, studied within 48hrs of life
2. The mother or caregiver of the child gives written informed consent for her/his child to participate in the study
3. Mothers, fathers and siblings (both HIV positive and negative) of HIV positive eligible infants, if they consent to enrolment in the study. The child may be enrolled in the study without participation of the mother or father if written informed consent for the child's participation is obtained.
5. The grandmother of eligible infants in cases where the mother was herself infected via mother-to-child transmission.
6. Mothers who had acute HIV infection during pregnancy (defined as a documented negative test during pregnancy) and their newborn tests negative by our point of care test (GenXpert qualitative HIV PCR).

Exclusion criteria:

1. Newborn infants not meeting the criteria specified above under 'inclusion criteria'
2. Mothers/fathers not meeting the criteria specified above under 'inclusion criteria'
3. The mother or caregiver of the child did not give written informed consent for his/her child to participate in the study

5. CLINICAL TRIALS [N/A]						
5.1 Has Medicines Control Council (MCC) approval been applied for?<br><br>[N/A – not a clinical trial]	Yes		No		N/A	X
5.2 Indicate current status of MCC approval application:						
5.3 Has this clinical trial been registered with the SA National Clinical Trials Register?	Yes		No		N/A	x
5.4 If "yes" to (5.3), please provide SANCTR registration number:						
5.5 If "no" to (5.3), PI hereby undertakes to register the trial with SANCTR after final ethics and MCC approval<br>Yes No xxN/						
5.6 Please provide the names of all members of the Data Safety and Monitoring Board (DSMB) (CLINICAL TRIALS ONLY)						
5.7 The PI hereby undertakes to ensure that all DSMB reports are forwarded to BREC for comment as soon as possible.<br>Yes No N/A						
5.8 Are any of the intended research participants in other research studies and/ or trials? *(If yes, describe in detail)*	Yes		No	X		
5.9 Is the PI presently involved in other research and/or clinical trial activities? *(If yes, please provide details and % time allocated to each)*<br><br>The research the PI is involved in currently is related to the two grants numbered 2-3 below, on which he commits 20hrs/week to each. Once grant number 1 starts in Feb 2015 the effort on grant 2 will be replaced by effort on this grant. This proposed study is the main focus of grant 1 and therefore the amount of time spent on this study by the PI is ~15hours/week.<br><br>Principal Investigator on the following grants:<br>1. Wellcome Trust Snr Investigator Award 01/02/15-31/01/20<br>Wellcome Trust £2,900,000 direct costs<br>Mechanisms of HIV non-pathogenicity and cure revealed in paediatric HIV infection<br><br>2. Wellcome Trust Programme Grant 01/07/12-30/06/15<br>Wellcome Trust £953,352 direct costs<br>Optimising CD8+ T cell responses in paediatric HIV infection<br><br>3. RO1-AI046995-12 (Goulder) 01/07/11-30/06/15<br>NIH/NIAID $1,939,716 total costs<br>Optimising CD8+ T-cell responses against C clade HIV infection in SubSaharan Africa	Yes	X	No			

5.10 Has the funder imposed any restrictions on PI regarding publication of study results?<br>If **Yes**, give details:	Yes		No	x	

**6. POTENTIAL RISKS OR DISCOMFORT**					
6.1 Can the project have any potential risks or discomfort on participants, members of the public, researchers, field staff or the physical environment?	Yes	**X**	No		

6.2 If "yes" to (6.1) indicate, for each study group/arm, the potential additional risks as follows:
6.2.1 Biological risks: Phlebotomy may cause a small amount of pain or bruising at the needle insertion site. Rarely, people faint during or after phlebotomy. Very rarely, an infection occurs at the site. Blood volumes will not exceed the recommended guidelines
6.2.2 Psychological risks: For protection of patient confidentiality, each subject will be assigned a unique study ID number. The files that identify a participant based on their study ID number will be kept by the investigators in a locked room on the hospital grounds. No unique patient identifiers will be entered into the dataset. Forms will be kept in a locked file and data will be kept indefinitely (see below). All data will be stored on password protected, encrypted computers.

6.2.3 Social Risks: None
6.2.4 Legal risks: None
6.2.5 Financial risks: None, expenses will be reimbursed as specified
6.2.6 Other risks:
Genetic Research Studies: Genetic research studies may create special risks to human subjects and their relatives. These involve medical, psychosocial and economic risks, such as the possible loss of privacy, insurability and employability, and limits on education options, and may create a social stigma. Knowledge of one's genetic make-up may also affect one's knowledge of the disease risk status of family members. To minimize the risks associated with genetic testing, no results will be filed in the subject's medical record and no research results will be given to subjects or healthcare providers.

6.3 Please detail steps that will be taken to minimise the risks indicated above:
6.3.1 Biological risks: Phlebotomy: The timing and frequency of blood collection as well as the quantity of blood collected will be recorded and closely monitored to avoid problems associated with phlebotomy.
6.3.2 Psychological risks: Anxiety: Research staff will explain the measures taken to ensure confidentiality of data.
6.3.3 Social Risks: None
6.3.4 Legal risks: None
6.3.5 Financial risks: Reimbursement/compensation as specified.
6.3.6 Other risks: Genetic Research Studies: To minimize the risks associated with genetic testing, no results will be filed in the subject's medical record and no research results will be given to subjects or healthcare providers

7. BIOLOGICAL SAMPLES					
7.1 Will human tissues (blood, blood products, gamete, gonads, oocyte, organs, flesh, bone, gland, skin, bone marrow or body fluids, waste materials such as urine and stools), microbial isolates and human genetic materials (DNA, RNA) be stored?	Yes	X	No		
7.2 If "yes" to (6.1), give details of storage facilities (name, location, conditions and duration of storage).<br><br>Samples of PBMC, plasma, DNA will be stored in the HPP facilities, DDMRI, UKZN. The duration of storage will be indefinite.					
7.2 Will human tissues, genetic materials and or microbial isolates be exported?<br><br>Samples of PBMC, plasma and DNA will be exported to collaborators (as detailed above) in Melbourne and Oxford for assays of viral reservoir, viral immunology, cytomegalovirus screening and genetic analysis	Yes	X	No		
7.3 If "yes" to (7.2), please attach current copies of export and import permits and International Aviation Clearance Certificates and a Materials Transfer Agreement (*see template on BREC website).* It is illegal to export human tissues and biological materials without an export permit (National Health Act, 2003).<br>Permission will be sought from UKZN BREC prior to the export of blood products. Thereafter, the necessary export / import permits will be applied for and a Material Transfer Agreement will be put in place. These documents will be submitted to the UKZN BREC prior to shipment.					
7.4 Please provide a rationale for export of biological materials (i.e. why the work cannot be done locally why local capacity cannot be upgraded)<br><br>As much of the work that can be done locally will be done locally within the HPP or with local collaborators. Collaborators overseas will be training personnel working on the project to undertake the majority of the assays to measure viral reservoirs. However a minor subset of the assays requires facilities and expertise not currently available locally and for this reason there is a need for export of certain samples for some of the projected work.					
7.5 Conflict of Interest:<br>Investigators should have no undisclosed conflict of interest with their study collaborators, sponsors or participants. Conflicts can arise, for example, when a commercial or other sponsor may not wish research results detrimental to their corporate image/interest to be disclosed, especially when the investigator is being remunerated by the sponsor for the research in question; or when an investigator has a vested interest in, or is an employee/shareholder/director in the sponsor's corporate entity. Conflicts of interest can also arise when an academic supervisor is also a co-investigator on a study with a student. Investigators should note that the duty to disclose a conflict of interest to BREC begins during application for ethical approval and continues until the research in question is complete and the research results are submitted to the sponsor/published (if applicable).<br><br>If the investigator(s) has/have/foresees any such conflict of interest, please provide details here:<br><br>Nil foreseen.					

8. GENERAL
8.1 Indicate, for each study group, the likely additional, i.e., over and above standard of care:<br>8.1.1 Duration of hospital stay (days): nil<br>8.1.2 Outpatient attendances (number): see Fig 1 above for schedule of out patient appointments<br>8.1.3 Laboratory services used, including those appointed by the sponsor (name and location): Global Laboratories, Durban, for viral loads and CD4 counts; HPP laboratories for research work as detailed above.<br>8.1.4 Type of samples and volumes to be drawn: see fig 1 for schedule of blood samples and volumes to be drawn.<br>8.1.5 Which laboratory services will be used? As above, Global Laboratories will be used for viral load and CD4 count measurements.<br>8.1.5.1 Has a preliminary agreement been reached with laboratory service providers? Yes.

Yes X		No	

If Yes, attach letter of confirmation. Please see letter of confirmation from Global Laboratories.

8.2 Has the nursing team who will be involved in the study been informed of the study and the nursing involvement which will be required?
(If no, please explain; other, please specify)

Yes		No	
X			

So far we have discussed the project with the paediatric and nursing/midwifery staff at Edendale Hospital, in order to introduce the proposed study and to determine the feasibility of the project at this site. The lead clinician with whom we are collaborating at Edendale hospital is Dr Roopesh Bhoola, and the additional paediatrician with whom we are collaborating at Edendale is Dr Malini Krishna. However it is important to stress that no extra work will devolve upon the nurses and paediatricians already working at the proposed study sites – all the extra work involved in the study will be undertaken by research personnel employed by the research study itself. Subsequently we have formed relationships with key paediatricians and their nursing staff teams, Dr Jeroen Van Lobenstein, Dr Constance Kapongo and Dr Kogie Chinniah at Stanger, Lower uMfolozi War Memorial and Mahatma Gandhi Memorial hospitals respectively to enable to study to run effectively and harmoniously with the Department of Health activities.

8.3 In the case of participants drawn from patient populations, indicate, in respect of each sub-group, how management differs from that usually offered to patients with similar conditions.

The study will adhere exactly to South African treatment guidelines in terms of the ART received. The only change will be that we will undertake the early testing of 'at-risk' infants. Once a diagnosis of HIV infection will be made, the treatment and management will be the same, other than for the scheduling of outpatient appointments (see Fig 1 above) that may differ somewhat from follow up scheduling at each study site. The HIV exposed and unexposed negative control groups will provide a blood sample, which is additional to standard management.

8.4 In the case of community based studies, explain what consultation is planned within the community at the following stages:
8.4.1 Preparation N/A
8.4.2 Implementation of the study and N/A
8.4.3 Dissemination of the results thereafter N/A

8.5 State the expected benefits arising from this study under the following headings:
8.5.1 <u>Possible direct benefits to study participants:</u> 'At risk' infants are by definition at risk of in utero and of intra partum HIV infection. Early diagnosis and ART initiation has been shown to be of considerable benefit in reducing mortality and morbidity in infected infants (Violari *et al, New Eng J Med*, 2008) and thus any infants in whom HIV infection is detected at birth and who receives ART within 48hrs of birth will benefit from this study.
8.5.1.1 <u>Clinical care</u>: If these studies can demonstrate the feasibility of this approach to early diagnosis and treatment, this would provide a rationale for this approach to be employed more widely elsewhere.
8.5.1.2 <u>Public health</u>: as above under 'Clinical care;.
8.5.1.3 <u>Financial</u>: N/A
8.5.1.4 <u>Prospects of tested intervention being available to the study population if proven effective</u>: N/A
8.5.1.4 Other (Specify):

8.5.2 <u>Specify the Indirect benefits arising from this study:</u> The research studies being undertaken in parallel may demonstrate that a proportion of infants receiving ART so early in life may develop so-called 'functional cure' of HIV over the course of the next 24 months or more on ART. This early intervention in babies may therefore provide insights into how functional cure may be achieved not only in newborns but also in older children and adults infected with HIV. In South Africa more than 20% of infants are HIV exposed uninfected (HEU). In 2015, excess mortality in HEU infants

accounted for almost 14% of mortality in all infants which accounts for approximate 2 times the mortality associated with infant HIV infection. (Slogrove et al. Reviews in Antiviral Therapy and Infectious Diseases 2017). Our study of antiviral immune responses in HEU and HUU control infants may reveal potential biological explanations for HIV exposure's significant contribution to South Africa's infant mortality rate. If we are able to characterize the suspected increased rate of in-utero HIV infection in females it may reveal further insight into the acquisition and pathogenicity of HIV in fetuses.

8.6 Describe the intended strategy for dissemination of study results:

8.6.1 To the scientific community: Through oral and written contributions to meetings and conferences and to scientific journals

8.6.2 To research participants: The same oral and written publications will be available to study participants.

8.6.3 To the general public (if applicable): The same oral and written publications will be available to the general public.

8.6.4 Other: Specify:

9. RESEARCH DATA/SAMPLES

9.1 Please explain where the data/samples will be stored and how long they will be stored for?

The samples will be stored in the HPP laboratories as described above, indefinitely. Data will be stored on password controlled computers, and/or stored on paper in files within locked cabinets and stored for the duration of the project (5 years in the first instance, potentially longer depending on further funding)

9.2 Will data/samples be destroyed after analyses? *(If no, please explain)* No, as it is envisaged that further valuable analyses may be undertaken at any point in the future.	Yes		No	X	

10. INFORMED CONSENT: GIVEN TO PARTICIPANTS

See attached

See SAMPLE INFORMATION SHEET AND CONSENT FORM ON UKZN BREC WEBSITE at *http://research.ukzn.ac.za/Libraries/Notices2011/BREC_Informed_consent_form_sflb.sflb.ashx*

Other consent forms are acceptable provided that they contain at least the essential elements outlined in the current UKZN BREC Terms of Reference (ToR) and Standard Operating Procedures (SoP) available at *http://research.ukzn.ac.za/Research-Ethics/Biomedical-Research-Ethics.aspx*

If necessary, information sheets and consent forms, after ethics approval of the English version, must be translated into appropriate local languages and submitted to BREC for further approval prior to implementation, with a copy of the translator's certificate, and back translations if applicable.

The correct and complete contact details for the UKZN Biomedical Research Ethics Committee should be in the information sheets and consent forms as follows:

BIOMEDICAL RESEARCH ETHICS ADMINISTRATION
Research Office, Westville Campus
Govan Mbeki Building
University of KwaZulu-Natal
Private Bag X 54001, Durban, 4000
KwaZulu-Natal, SOUTH AFRICA
Tel: 27 31 2602486 - Fax: 27 31 2604609
Email: BREC@ukzn.ac.za

11. QUESTIONNAIRES: GIVEN TO PARTICIPANTS

Provide 25 copies of all questionnaires, interview guides, data collection sheets etc.

List all such attachments here:

12. DECLARATION OF PRINCIPAL INVESTIGATOR

Conflict of Interest:

I declare that all potential conflicts of interest regarding my application for ethics approval to conduct this study have been declared in accordance with UKZN and BREC Terms of Reference and Standard Operating Procedures.

Oversight of study: Will this study be overseen by a professional Clinical Research Organisation or study sponsor?

Yes		No	X

If Yes, please give details:

Undertaking:

I understand and accept that I will be required to submit a yearly recertification application, failing which authorisation to continue the study lapses. Progress reports may be required more frequently depending on level of risk and other factors – this will be detailed in the BREC approval letter. Where applicable, all reports from the Data Safety Monitoring Boards (or similar committees) will be provided to the Biomedical Research Ethics Committee within 7 days.

I undertake to request permission for any changes/amendments to the study from BREC in advance of implementing any such changes, unless they are emergencies required to prevent harm or save life. In such cases BREC must be notified urgently.

I agree to provide monitoring data if and when required.

I expect the project to be completed by **DATE**………Feb 1st 2020…………………………

I agree to abide by the guidance contained in the SA Department of Health (2004) Ethics in Health Research: Principles, structures and processes and the (2006) South African Good Clinical Practice Guidelines and the current UKZN Biomedical Research Ethics Committee Terms of Reference and Standard Operating Procedures. These are available at *http://research.ukzn.ac.za/Research-Ethics/Biomedical-Research-Ethics.aspx*

I understand and accept that all information pertaining to this application is a true reflection of the project proposed and I take full responsibility should there be any transgression.

SIGNATURE OF PRINCIPAL INVESTIGATOR…

FULL NAME OF PRINCIPAL INVESTIGATOR…Philip Jeremy Renshaw Goulder………

DATE……7th October 2014…………..

12. DECLARATION AND APPROVAL FROM SUPERVISOR AND CO-SUPERVISOR (if applicable) *(I HAVE READ AND CHECKED THE PROPOSAL AND IT IS READY FOR SUBMISSION;*

Remarks:

SIGNATURE OF SUPERVISOR ………...………………………………

FULL NAME OF SUPERVISOR…………………………………………

DATE………………………………………..

SIGNATURE OF CO-SUPERVISOR ………..…………………………………

FULL NAME OF CO-SUPERVISOR…………………………………………

DATE………………………………………..

If applicable, attach a signed copy of the Supervision Agreement between the student, supervisor and any co-supervisor.

13. DECLARATION AND APPROVAL OF LINE MANAGER

(Must include verification of interdepartmental agreements and co-operation)

Remarks:

SIGNATURE OF LINE MANAGER ……………………………………

FULL NAME OF LINE MANAGER……………………………………

DATE………………………………………..

NB: If applicant is ACADEMIC LEADER/DEAN/HOS, the ACADEMIC LEADER'S/DEAN'S/HOS's Line Manager (DVC) must sign.

SIGNATURE OF ACADEMIC LEADER's/ HOS's/DEAN's Line Manager……………………………………

FULL NAME OF ACADEMIC LEADER's, HOS's/DEAN's Line Manager……………………………………

DATE………………………………………..
